# Cytochrome c: Using Biological Insight toward Engineering an Optimized Anticancer Biodrug

**DOI:** 10.3390/inorganics9110083

**Published:** 2021-11-16

**Authors:** Louis J. Delinois, Omar De León-Vélez, Adriana Vázquez-Medina, Alondra Vélez-Cabrera, Amanda Marrero-Sánchez, Christopher Nieves-Escobar, Daniela Alfonso-Cano, Delvin Caraballo-Rodríguez, Jael Rodriguez-Ortiz, Jemily Acosta-Mercado, Josué A. Benjamín-Rivera, Kiara González-González, Kysha Fernández-Adorno, Lisby Santiago-Pagán, Rafael Delgado-Vergara, Xaiomy Torres-Ávila, Andrea Maser-Figueroa, Gladimarys Grajales-Avilés, Glorimar I. Miranda Méndez, Javier Santiago-Pagán, Miguel Nieves-Santiago, Vanessa Álvarez-Carrillo, Kai Griebenow, Arthur D. Tinoco

**Affiliations:** 1 Department of Chemistry, University of Puerto Rico, Río Piedras Campus, Río Piedras, PR 00931, USA;; 2 Department of Chemistry, University of Puerto Rico, Humacao Campus, Humacao, PR 00792, USA;

**Keywords:** cytochrome c, therapeutic proteins, smart drug delivery systems, intrinsic apoptosis

## Abstract

The heme protein cytochrome c (Cyt c) plays pivotal roles in cellular life and death processes. In the respiratory chain of mitochondria, it serves as an electron transfer protein, contributing to the proliferation of healthy cells. In the cell cytoplasm, it activates intrinsic apoptosis to terminate damaged cells. Insight into these mechanisms and the associated physicochemical properties and biomolecular interactions of Cyt c informs on the anticancer therapeutic potential of the protein, especially in its ability to subvert the current limitations of small molecule-based chemotherapy. In this review, we explore the development of Cyt c as an anticancer drug by identifying cancer types that would be receptive to the cytotoxicity of the protein and factors that can be finetuned to enhance its apoptotic potency. To this end, some information is obtained by characterizing known drugs that operate, in part, by triggering Cyt c induced apoptosis. The application of different smart drug delivery systems is surveyed to highlight important features for maintaining Cyt c stability and activity and improving its specificity for cancer cells and high drug payload release while recognizing the continuing limitations. This work serves to elucidate on the optimization of the strategies to translate Cyt c to the clinical market.

## 1. Introduction

Cancer ranks as a leading cause of global death and it is predicted that the burden of cancer incidence and mortality will continue to rise [1]. While it was estimated that ~19.3 million new cases and ~10.0 million deaths would occur in 2020 [1], the COVID-19 pandemic with its near global lockdown and requirement for physical distancing has severely impacted cancer diagnosis and treatment [2–4]. The ramifications of this disruption are certain to increase the number of cancer incidence and of the detection of cancer cases at stages more advanced than normal, and will present further complications to treatment, especially given the existence of a new pool of cancer patients: the COVID-19 and post-COVID-19 infected patient [2–4]. More than ever, there is a tremendous need to revise medical protocols to meet a higher standard of cancer care that can withstand the ongoing challenges imposed by COVID-19 and that can be adaptable to potential challenges of future pandemics. Improving cancer treatment regimens is at the apex of this need. There have been tremendous breakthroughs in cancer research discoveries [5–8], including a better understanding of the diverse microtumor environment and of the origins and distinct molecular profiles of specific cancer types, the identification of new druggable targets and vast drug delivery methods, and the clinical trial testing of new technologies such as CRISPR-Cas9 gene editing for cancer treatment. The application of all of this quickly developing knowledge is trailing behind. Nonetheless, given the pressing current needs, there is hope for a dynamic shift in translational cancer research.

Chemotherapy, the strategy of killing tumor cells, inhibiting their growth, or a combination of both, and proliferation of tumor cells by chemical drugs, was once the only cancer drug therapy. Today, chemotherapy, alongside surgery and radiation, remains one of the most common approaches to cancer treatment. Most chemodrugs are small molecules and are typically administered in combination with other chemodrugs. Despite their wide use, chemodrugs have major drawbacks stemming from their inability to distinguish between cancer and normal cells and thus they can result in toxicity and dose-limiting side effects. Given their general genotoxic behavior, cancer cells can evolve to resist the effects of the drugs, to inhibit their intracellular accumulation, or a combination of both. This process is termed acquired resistance. To circumvent the limitations of standard chemotherapeutics, targeted therapeutic drugs are emerging as prime cancer treatments. In this treatment space, the use of small molecules, namely protein inhibitors, remains quite pertinent [9]. Therapeutic biomacromolecules are also gaining a strong foothold.

**Figure 1.**
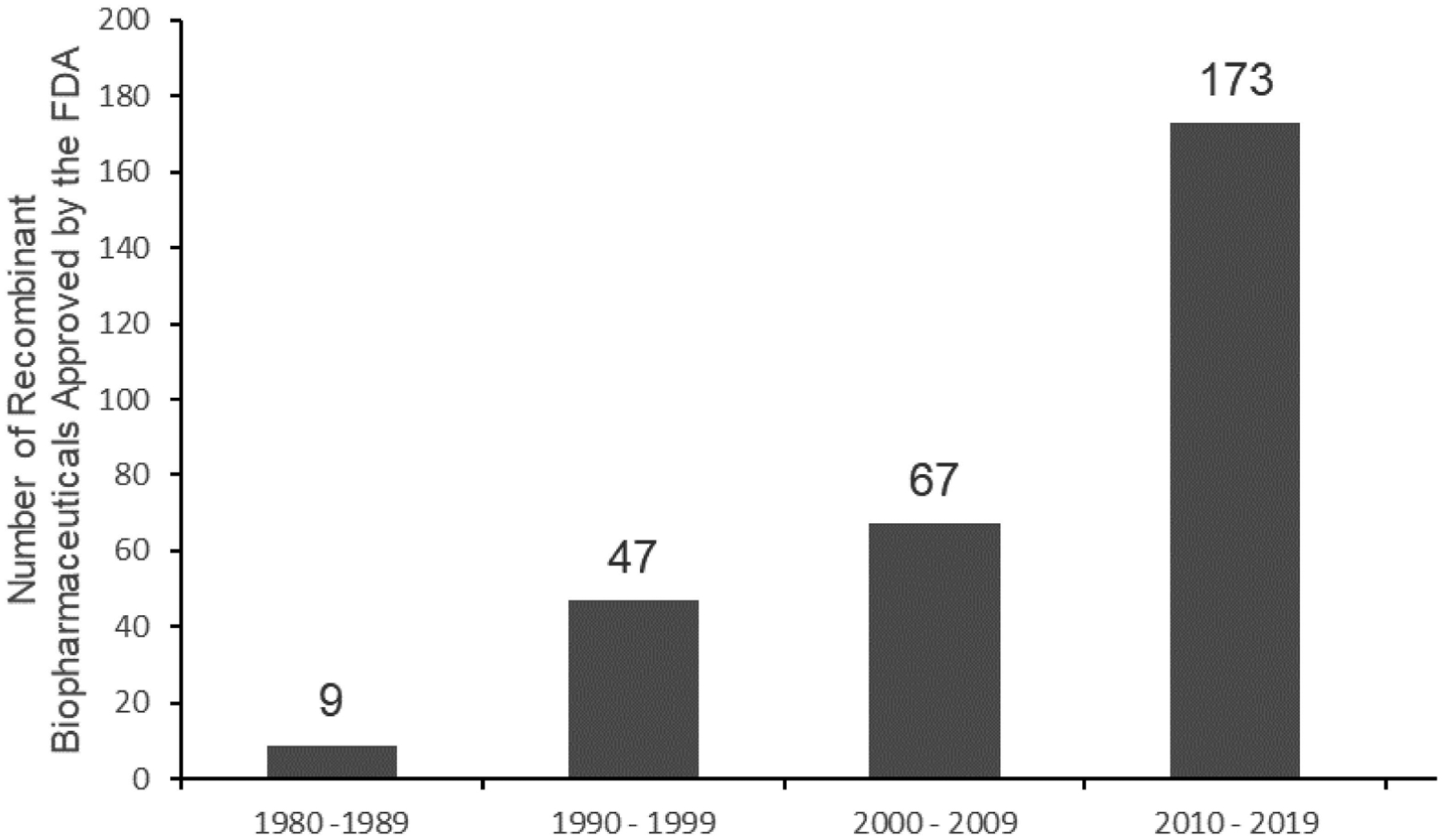
The number of FDA-approved recombinant biopharmaceuticals throughout decades. The content was taken from ref. [16].

Therapeutic proteins, which are engineered in the laboratory for pharmaceutical use, have gained much attention for their clinical utility due to their implication in dynamic and vital processes in the body [10–12]. They are counted among the fastest growing pharmaceuticals. Developing proteins as drugs is not trivial for many reasons. They are susceptible to proteolytic breakdown and cannot be taken orally due to poor stability in the digestive system. Some proteins are cell impermeable or do not have cell membrane receptors to facilitate the cell entry, which are specific to certain cell types. Nonetheless, therapeutic proteins are sought because unlike the small-molecule drugs, they can be very selective for specific disease hallmarks [13]. Initially, proteins were isolated from humans, animals, and plants. Insulin, the first therapeutic protein approved by the United States Food and Drug Administration (FDA), was primarily isolated from animal pancreases and administered to diabetic mellitus patients [14]. Despite the therapeutic benefits of these isolated proteins, certain complications, including pathogenic contamination and the growing demand for higher protein yield, have created the need for other ways to produce them. With the development of molecular biology techniques, in particular DNA recombinant technology, the first human recombinant therapeutic insulin was produced and approved by the United States FDA in 1982. The recombinant human insulin overcame most of the drawbacks that limited the use of the animal derived insulin [11,14] and thereby production of recombinant expression of therapeutic proteins was embraced by the industry (Figure 1). Data by the National Center for Biotechnology Information (NCBI) indicate that, as of the 1980s, at least 239 protein and peptide therapeutics and their 380 drug variants have been approved by the FDA [15]. Therapeutic proteins include antibodies, anticoagulants, blood coagulation factors, bone morphogenetic proteins, engineered protein scaffolds, enzymes, fragment crystallizable fusion proteins, growth factors, hormones, interferons, interleukins, and thrombolytics [14].

Recombinant proapoptotic proteins are an emerging class of cytotoxic biopharmaceuticals with high potential as anticancer agents and as alternatives to conventional small molecule drugs [12,17–20]. Apoptosis is a programmed cell death mechanism that is crucial for maintaining tissue homeostasis in multicellular organisms by balancing cell proliferation and cell death [21–23]. The heme protein cytochrome c (Cyt c) is one of the most promising proapoptotic proteins for cancer therapy. Its participation in apoptosis in the cytosol is juxtaposed with its pivotal role at the mitochondrial inner membrane in cellular respiration as part of the electron transport chain (ETC). These functions are highly regulated by mitochondria and the protein’s heme cofactor. This review article explores various layers of the utility of Cyt c in cancer research by examining key biological insights of how the protein functions. The protein is revealed to be a possible biomarker for the detection of cancer and to be central to the anticancer mechanism of the action of several small molecule anticancer drugs. Furthermore, this review serves to provide an overview of the efforts of numerous research teams to develop Cyt c as an anticancer drug by establishing a proficient drug delivery system and formulation to overcome the cell impenetrability of the protein and to enable specificity for cancer cells. By learning from these efforts and highlighting important structural details of the protein essential for activity, the ultimate objective is to shed light on an optimal approach to translate Cyt c to the drug market.

## 2. Examining the Biological Functions of Cyt c

### 2.1. Evolutionary Insight of Cyt c

**Figure 2.**
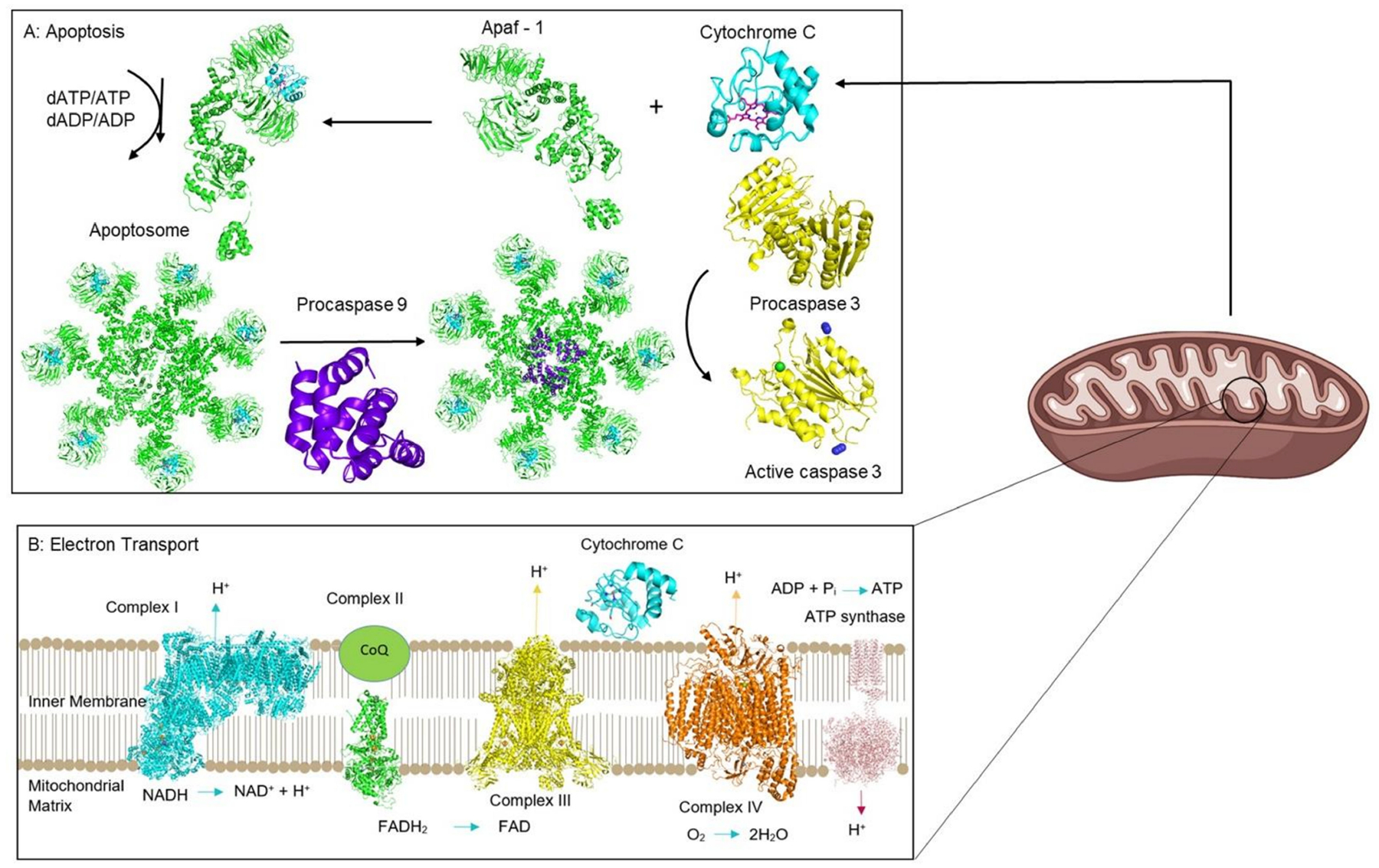
Major roles played by Cyt c. Cyt c interacts with several protein as it participates in intrinsic apoptosis in the cytoplasm (**A**) and cell respiration in the ETC of mitochondria (**B**). PDB codes: 6ZKI, 1ZOY, 3CX5, 5Z62, 1QO1.

**Figure 3.**
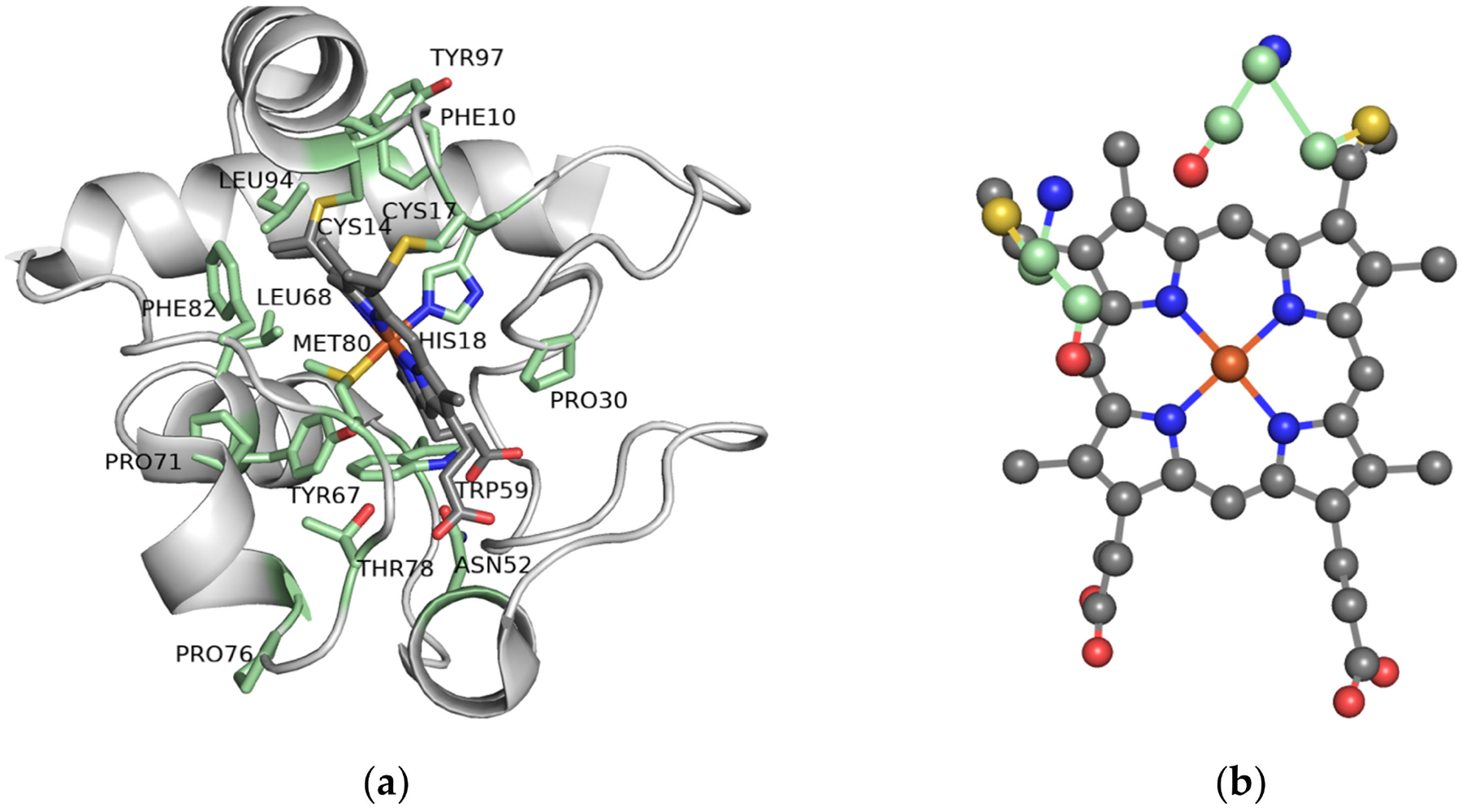
Horse heart Cyt c. (**a**) Three-dimensional structure of horse heart Cyt c in which the highly conserved residues are highlighted in green. (PDB: 1HRC); (**b**) The heme group and its covalent attachment to Cys14 and Cys17 (in yellow).

**Figure 4.**
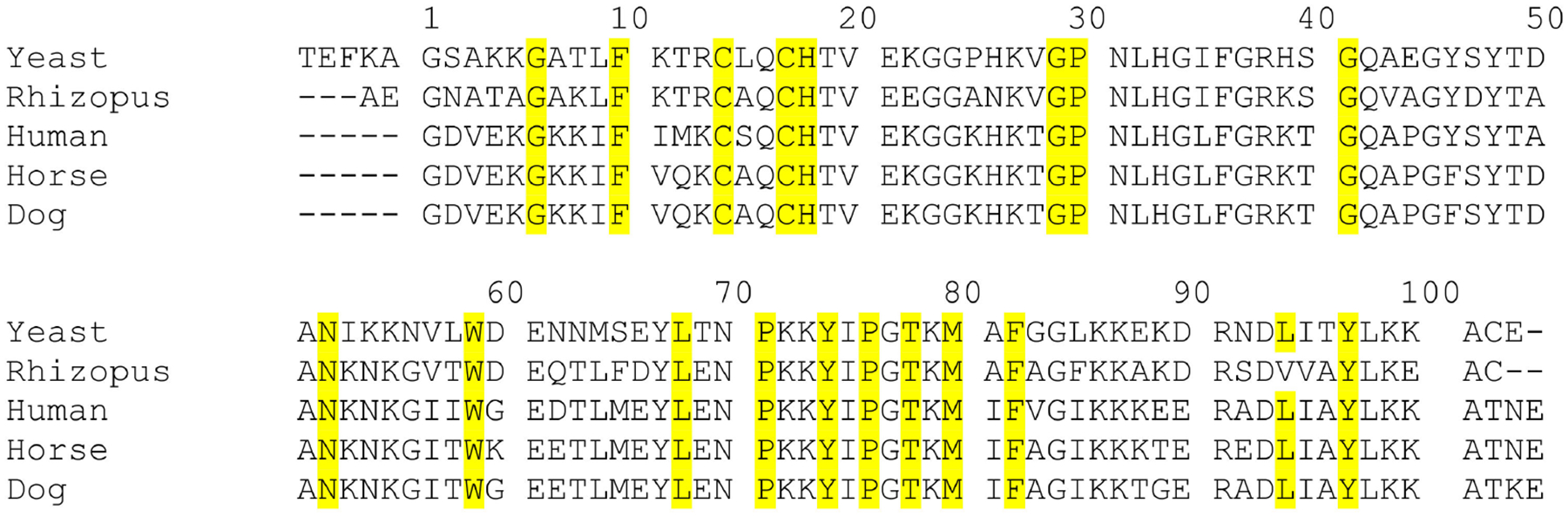
Multiple sequence alignment of Cyt c from different eukaryotic species in which select conserved residues are highlighted in yellow. For simplicity, only a portion of the Cyt c sequence is presented. Protein accession numbers (P00044 for *Saccharomyces cerevisiae* or Baker’s yeast, I1C9J3 for *Rhizopus delemar*, P99999 for Homo sapiens, F7D4V9 for Equus caballus, P00011 for *Canis lupus* familiaris) were retrieved from the NCBI data base and the sequence alignment was conducted using Clustal Omega by EBI.

Cyt c is a ubiquitous protein that plays a key role in the ETC, in serving as a redox agent, in regulating reactive oxygen species (ROS), and in inducing apoptosis in eukaryotes (Figure 2). It is composed of about 104 amino acids with a heme prosthetic group covalently attached to two cysteines (Cys14 and Cys17) and the Fe center ligated by two protein residues (His18 and Met80) at its axial positions (Figure 3). Cyt c’s structure is characterized by being highly conserved across different species of organisms. Throughout evolution, the sequence of this protein has not undergone considerable mutations and therefore the domains and residues that are crucial for its function and integrity are preserved (Figure 4) [24].

**Figure 5.**
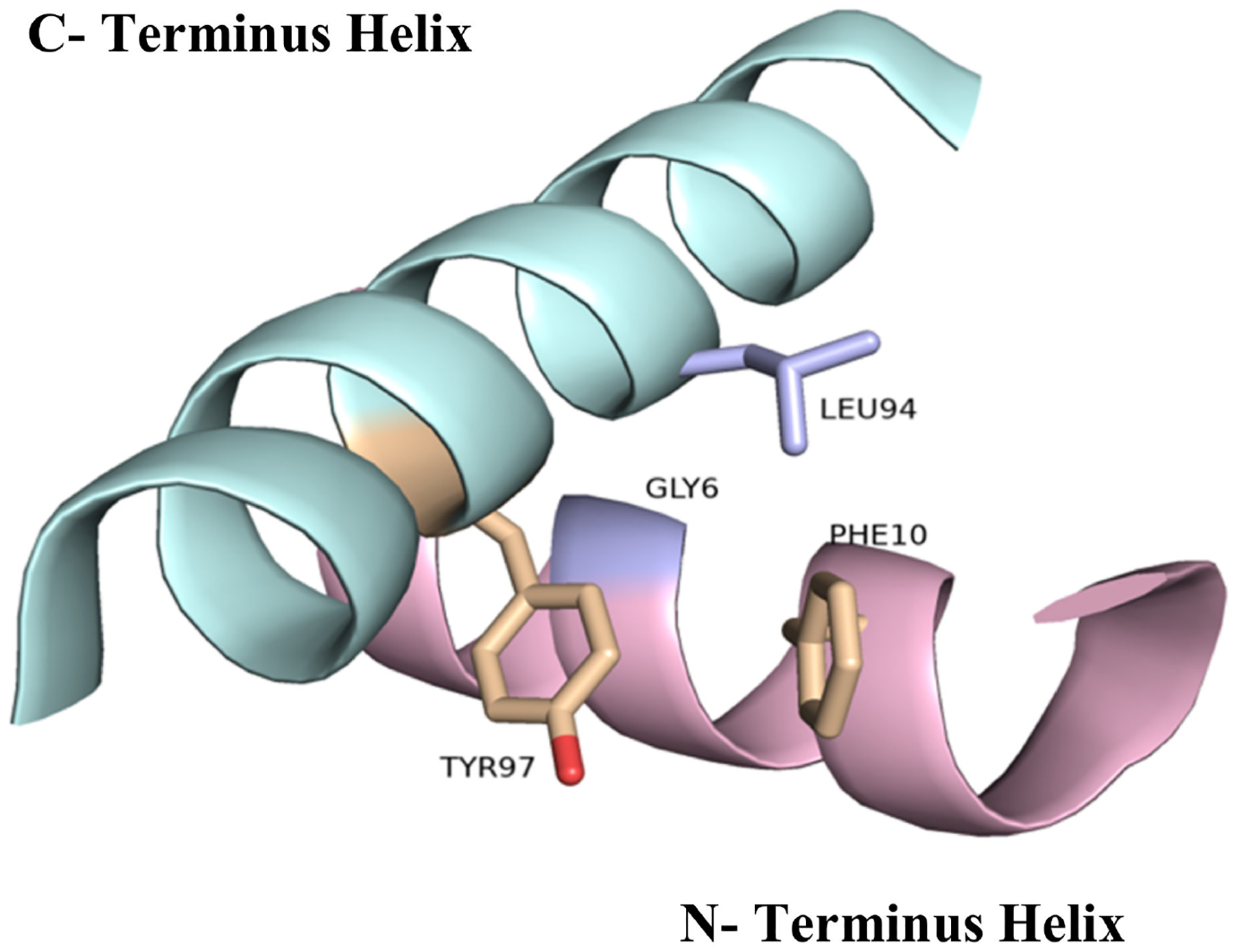
Interface formed between the N and C terminal helices of Cyt c showing key conserved residues in stick model. As shown here, Tyr97 and Phe10, which interact with each other through an aromatic–aromatic interaction, while Leu94 is in close proximity to Gly6 (PDB: 1HRC).

Several residues including Gly6, Phe10, Cys14, Cys17, His18, Gly29, Pro30, Gly41, Asn52, Trp59,Tyr67, Leu68, Pro71, Tyr74, Pro76, Thr78, Met80, Phe82, Leu94 and Tyr97 have been shown to be ubiquitous in the sequence of Cyt c among different species [25]. These highly conserved residues play a crucial role in the preservation of the tertiary structure of the protein and are essential for its activity. For example, it has been shown that the N and C termini of Cyt c fold into alpha helices that interact perpendicularly with each other (Figure 5). This motif is thought to be of considerable importance in the early stages of the folding process of this protein. The helices interact with each other as soon as the heme is covalently bound to the polypeptide via thioether bonds to Cys14 and Cys17 at the end of the N-terminal helix, meaning that this interaction directs subsequent folding steps in the protein. Therefore, alterations on the interface of the N and C terminal helices can directly affect the folding and heme attachment process of Cyt c and interfere with its function. Highly conserved residues such as Gly6, Phe10, Leu94, and Tyr97 are found to establish key electrostatic interactions to allow the association of the N and C terminal helices. Leu94 and Tyr97 from the C-terminal helix interact with Gly6 and Phel0 from the N-terminal helix, respectively, to bring the helices together. It is worth mentioning that the importance of these residues and the reason for their preservation through evolution is not always associated with the stability of the terminal helixes or the folding kinetics. For instance, the conservation of Gly6 has been attributed to the limited space in this region of the protein that does not accommodate bulkier residues [25].

**Figure 6.**
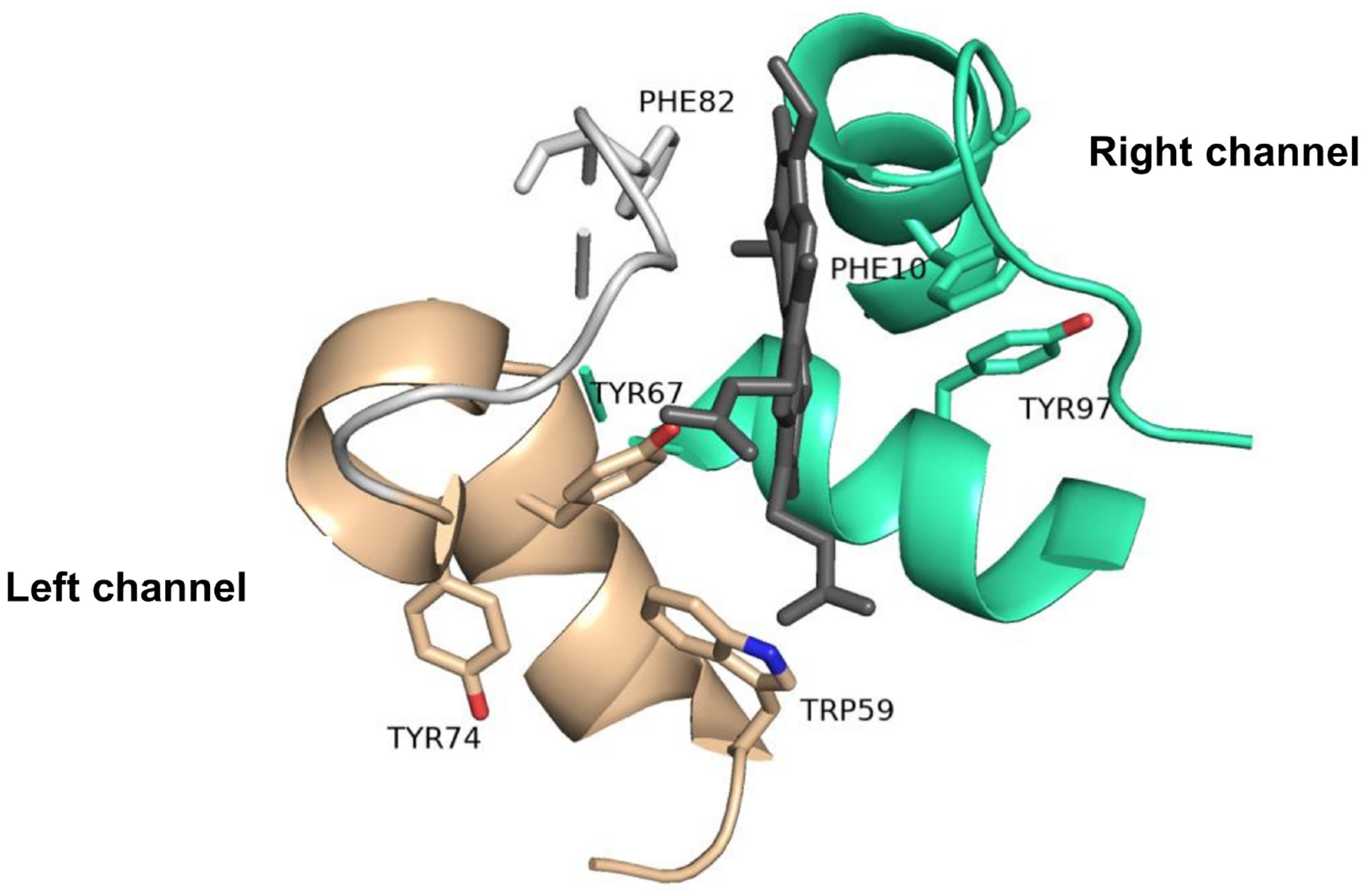
Left and right channels of Cyt c showing conserved aromatic residues that are in close proximity to the heme group and therefore are essential for the redox activity of the protein (PDB: 1HRC).

The binding of the heme group of Cyt c is crucial to its ability to transport electrons. The Fe ligation by His18 and Met80 modulates the interconversion of the Fe between the Fe^2+^ and Fe^3+^ oxidation states by ensuring a low-spin state of the ion and a low reorganization energy of the site [26]. This metal coordination enables an Fe^3+/2+^ redox potential (E_m,pH 7_ = 250 mV vs. standard hydrogen electrode) necessary for the protein to exert its functional role in mitochondrial respiration as an electron carrier between respiratory complexes III and IV (Figure 2) [27]. Coenzyme Q–cytochrome c reductase (respiratory complex III) catalyzes the transfer of electrons from ubiquinol to the oxidized form of Cyt c. After being reduced, each molecule of Cyt c carries one electron at a time to the next respiratory complex of the chain: the cytochrome–oxidase complex. This complex is the last enzymatic unit of the ETC and catalyzes the transfer of four electrons from four Cyt c molecules to molecular oxygen. Aromatic amino acid residues such as Trp59, Tyr67 and Phe82 are found in close proximity to the heme group and play a key role in the stability and modulation of the redox potential of this catalytic center [24,25]. They belong to an essential region of the protein called the left channel (Figure 6). Biochemical studies suggest that this channel is directly involved in the reduction mechanism of the protein and facilitates the association of Cyt c with the cytochrome–reductase complex. Similarly, Phe10 and Tyr 97 have been described to be associated with another important region of Cyt c known as the right channel (Figure 6), which together with the heme cleft, is responsible for the interaction and reaction with the cytochrome–oxidase complex [24].

The distribution of electrically charged residues is also conserved throughout evolutionary history and is important for Cyt c’s role in electron transport. Most of the electrically charged residues of Cyt c are found on its surface. A total of nineteen lysine residues are distributed in the vicinity of the left and the right channel of the protein, while the region between both channels holds a series of acid residues. This distribution of positive charges in the edge of the channels and negative charges in the central region is believed to be essential for the association of Cyt c with its oxidase [24].

Evolutionary conservation exists with respect to the apoptotic function of Cyt c. Apoptosis involves a process of signaling and responses to stimuli that results in programmed cell death. This mechanism of population control of cells is crucial in optimal development and tissue homeostasis. Cyt c assists in the apoptosis process through an interaction with the regulatory protein apoptotic protease activating factor-1 (Apaf-1). In general terms, Cyt c is released from the mitochondria into the cytosol after a signaling process, in which it binds to Apaf-1, causing conformational changes in this protein, allowing the apoptosome formation and protease caspase activation. It is important to note that released Cyt c may sometimes participate in inducing cell differentiation [28]. Structural studies revealed that Cyt c’s Gly41 and Lys39 residues are in close proximity to each other and are involved in the Cyt c-Apaf-1 compound formation [29,30]. Gly41 is a key residue that has remained invariant throughout evolution in around 113 species of eukaryotes and it is believed to be crucial for optimal functioning and preservation of the structural integrity of Cyt c [31]. Mutations that may cause the substitution of this amino acid could lead to an alteration in the apoptotic activity of this protein. Morison et al. reported the first mutation identified in the gene that encodes Cyt c in humans in a family with autosomal dominant thrombocytopenia causing a Gly41-Ser41 substitution [32]. Structural analyses by X-ray crystallography revealed that this substitution does not induce a considerable alteration of the spatial conformation of Cyt c. The redox potential of the protein is not altered, and the mitochondrial oxygen consumption rate is unaffected. The Gly41-Ser41 substitution, however, caused a considerable increase in its apoptotic activity compared to the wild-type variant [32]. The G41S mutant is believed to be responsible for familial thrombocytopenia. Patients with this condition are otherwise healthy and live long lives, which suggests that this mutant variant of Cyt c does not have any considerable effect on the development of organs [32].

### 2.2. Classes of Cyt c

**Figure 7.**
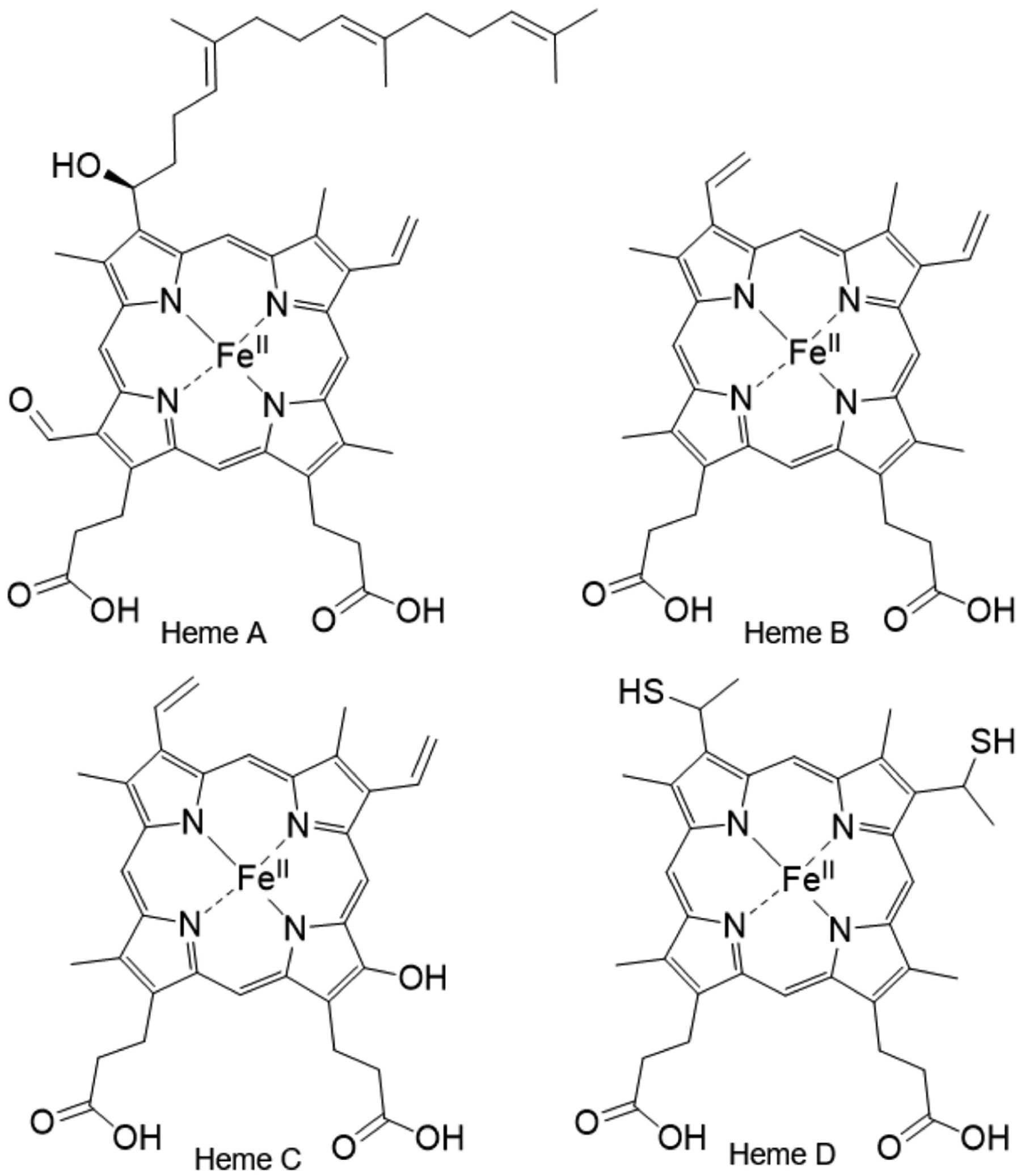
Structure of the heme types.

There are four main classes of Cyt c in mammalian organisms (I-IV), which are categorized based on the number of hemes, the position and identity of the axial iron ligands, and the reduction potentials [33,34]. The heme groups that can be present in Cyt c can be classified into types A–D depending on the substituents on the porphyrin ring (Figure 7) [35]. Cyt c are made of the type C heme, hence their name. Cytochrome a, b, and d contain heme types A, B, and D, respectively. Class I Cyt c proteins are small (8–12 kDa), water soluble, and possess a conserved binding motif in the N-terminal with a Cys-Xxx-Xxx-Cys-His sequence, in which the cysteines covalently bind to the heme group, the Xxx represent any amino acids, and the His serves as the axial ligand for the heme Fe. This class consists of five total α-helices. Class II Cyt c contains the Cys-Xxx-Xxx-Cys-His sequence motif near the C-terminus but differs in having only four α-helices and a left-handed twisted structure. Class III Cyt c include more than one heme group with bis-His ligation. Structural characteristics are two β-sheets and five α-helices. Lastly, Class IV Cyt c are large 40 kDa cytochromes and contain different prosthetic groups in addition to four Heme groups. Herein, the term Cyt c refers to the class 1 Cyt c present in eukaryotic mitochondria, particularly in mammals.

### 2.3. Cyt c as a Multifunctional Protein

Cyt c’s functions depend on its cellular localization and its structure as influenced by the conditions in which it operates. Thus, Cyt c can either be involved in processes that promote cellular life or that terminate it (i.e., apoptosis). Cyt c is a mitochondrial peripheral membrane protein, which mediates electron transfer between complexes III and IV of the respiratory chain. It is central to aerobic energy production. Adenosine triphosphate (ATP) synthesized in this process serves as an energy reservoir. The Cyt c structure was shown by Englander and collaborators to be the result of five cooperative folding–unfolding units called foldons that follow interdependent sequential stabilization [36–38]. One of the foldons consists of the loop formed by the residues 71–85, which includes the axial heme Fe binding residue Met80. This foldon is of very low stability due to the Fe-Met80 bond being relatively weak. The Met80 can be replaced by other residues, such as a Lys or a His, which results in major structural changes that alter the function of the protein. This substitution is observed for Cyt c molecules that interact with cardiolipin (CL, a phospholipid of the mitochondrial membrane). These Cyt c molecules develop peroxidase activity as a consequence of a bis-His coordination negatively shifting the heme Fe redox potential by approximately 350–400 mV. Due to this redox potential shift, Cyt c is able to catalyze cardiolipin peroxidation, which then perturbs the Cyt c-CL interaction and results in Cyt c release into the cytoplasm [39].

**Figure 8.**
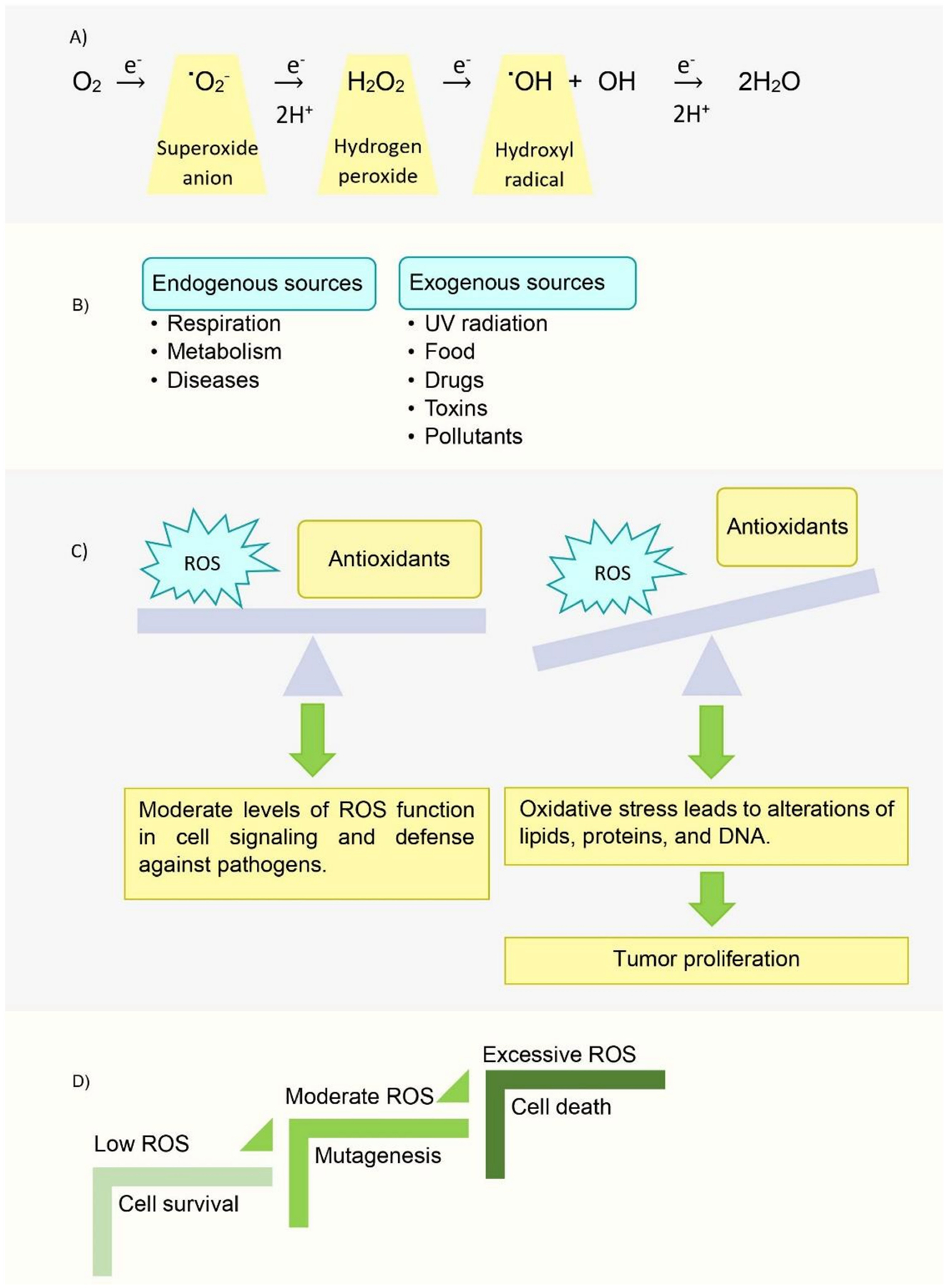
Reactive Oxygen Species. (**A**) Complete reduction of oxygen via ROS formation. (**B**) Sources of ROS production. (**C**) Balance of ROS and antioxidants in normal cells. (**D**) Levels of ROS in cancerous cells.

In healthy cells, Cyt c also serves as a detoxifying agent to dispose of ROS. ROS are products of oxygen metabolism in aerobic organisms, and they include superoxide, the hydroperoxyl radical, the hydroxyl radical, hydrogen peroxide and singlet oxygen. A main metabolic pathway through which ROS are produced in eukaryotes is the mitochondrial ETC. Electrons leak out of the ETC and are captured by molecular oxygen, forming the superoxide anion (Figure 8A). Then, mitochondrial superoxide dismutase (SOD) breaks these anions down to hydrogen peroxide [40]. ROS are produced endogenously in normal cellular processes and are essential in some biological functions: the immune system produces them to kill pathogens and some ROS are used in cell signaling (Figure 8B). However, due to their high reactivity, they may become harmful to the cell in large quantities, as they are capable of oxidizing species, such as lipids, proteins, and DNA (Figure 8C). For this reason, the cell has mechanisms to control ROS levels, such as their reduction by antioxidants, for example, SOD. When the amount of ROS is increased excessively, or when the amount of antioxidants is reduced, this can result in oxidative stress. This imbalance may be related to mutation, carcinogenesis, degenerative diseases, and the aging process. In the same manner as healthy cells, cancerous cells need ROS for normal function, but they are also susceptible to deleterious effects from excessive ROS production: these species are involved in the apoptosis of cancerous cells [41] (Figure 8D). Modulation of ROS levels has been investigated as a possible therapeutic treatment of cancer: drugs that increase ROS production may help to induce apoptosis of cancerous cells [41–43] and drugs that scavenge ROS species may help to combat oxidative stress, which would reduce carcinogenesis [41].

Cyt c operates as a superoxide radical and H_2_O_2_ scavenger; in the first case, the oxidized protein regenerates O_2_ within the inner mitochondrial membrane space, removing the unpaired electrons from superoxide, while, in the second case, the conversion of H_2_O_2_ is managed by Cyt c both in the reduced and in the oxidized state [44]. The ability of Cyt c to act as an ROS scavenger gives it an active role against oxidative stress not only in the cellular defense against cancer, but also against other diseases that are associated with an increase in ROS production, such as ischemia. In fact, the amount and the redox state of Cyt c have been shown to influence mitochondrial H_2_O_2_ production. As an example, Pasdois et al. showed that a decrease in Cyt c levels accounts for an increase in ROS [45].

**Figure 9.**
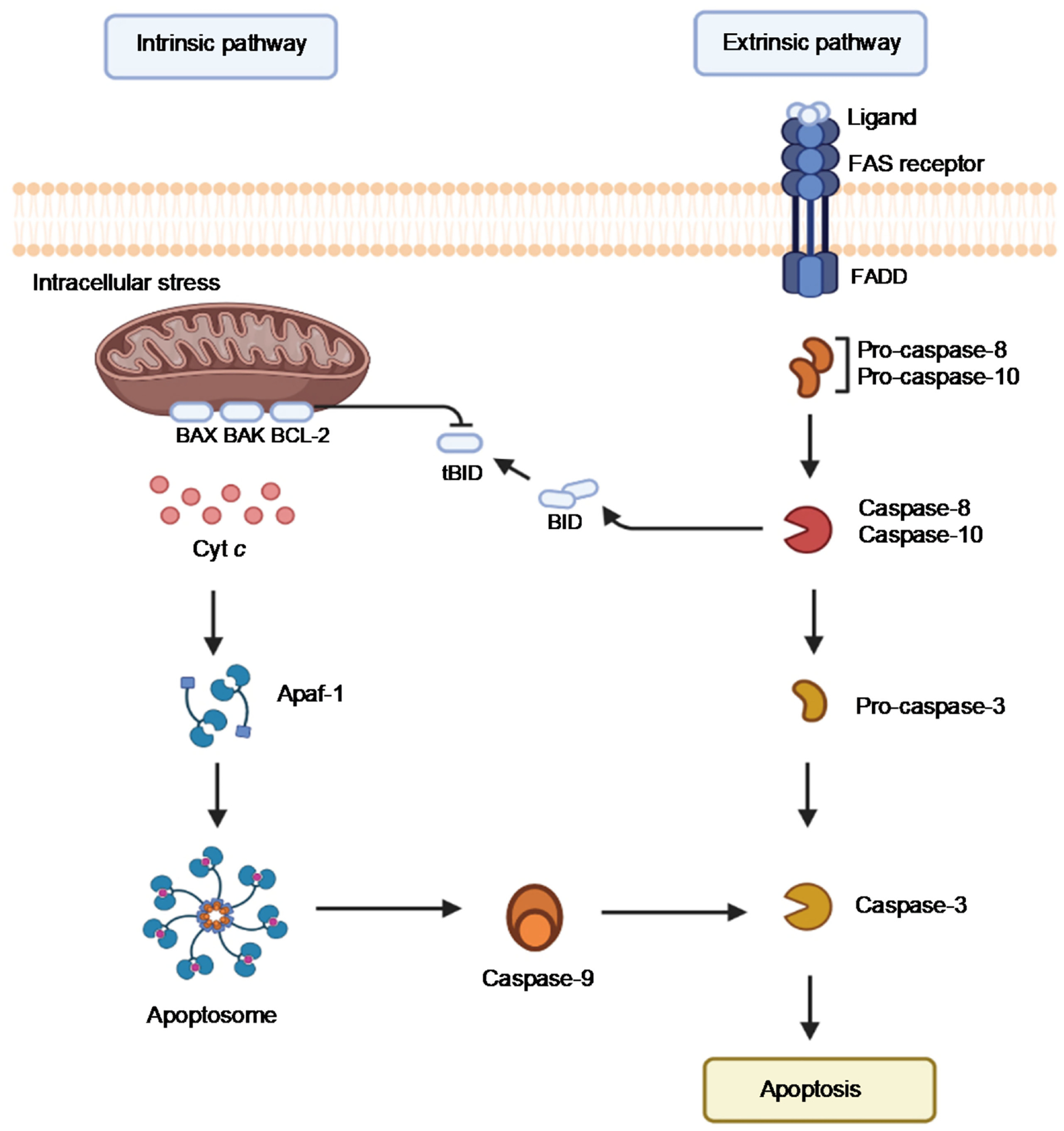
Schematic view of the apoptotic process promoted by the ‘intrinsic’ and the ‘extrinsic’ pathway. (Image was created using BioRender.com).

Under cellular oxidative stress due to excess ROS production, Cyt c takes on the role of inducing apoptosis. Apoptosis is a genetically directed process of cell self-destruction that occurs in multicellular organisms, which can take place either through the extrinsic or the intrinsic pathway (Figure 9) [46]. The extrinsic pathway is initiated by an extracellular ligand binding to the FAS receptor; a transmembrane death receptor. The adaptor protein FADD next binds to the ligand–receptor complex, which then activates initiator caspases, caspase-8, and caspase-10. This process triggers the enzymatic cascade leading to cell death. The intrinsic pathway is activated by cellular harm, including DNA damage, ischemia, and oxidative stress [46]. Its primary function is to eliminate damaged cells [47]. The intrinsic pathway is controlled by members of the BCL protein family bound to the mitochondrial membrane. The BAX protein is an example of a pro-apoptotic regulatory protein whereas BCL-2 is an anti-apoptotic regulatory protein, which inhibits Cyt c release [48]. Upon mitochondrial membrane perturbation because of oxidative stress, Cyt c is detached from the inner mitochondrial membrane and released into the cytoplasm. It then forms a complex known as an apoptosome with Apaf-1 and the inactive form of caspase 9. The apoptosome hydrolyzes ATP, which in turn cleaves and activates caspase 9. Caspase 9 then activates caspases 3, 6, and 7, which triggers cell apoptosis.

### 2.4. Cyt c Phosphorylation as a Regulator of Cyt c Activity

Posttranslational modification (PTM) of Cyt c can greatly alter the protein’s bioactivity [49]. A new model has been proposed that Cyt c phosphorylation regulates the protein’s activity in the ETC and apoptosis [44]. Kalpage et al. modified protein purification protocols to preserve the in vivo phosphorylation of mitochondrial proteins, enabling the discovery of novel phosphorylation sites on both Cyt c oxidase (COX) and Cyt c in mammals. A total of five phosphorylation sites of Cyt c Tyr97, Tyr48, Thr28, Thr58, and Ser47 were outlined and functionally characterized. A total of four of the phosphorylation sites result in inhibition of the apoptotic functions of Cyt c, suggesting a cytoprotective role for phosphorylated Cyt c [44]. These phosphorylation sites are lost during stress conditions (e.g., ischemia). This results in maximal ETC flux during reperfusion-tissue injury, mitochondrial membrane potential hyperpolarization, excessive ROS generation, and apoptosis. Kalpage et al.’s new model proposes that the electron transfer from Cyt c to COX is the rate-limiting step of the ETC, regulated via phosphorylation of Cyt c. This regulation may be dysfunctional in disease conditions such as ischemia-reperfusion injury and neurodegenerative disorders through increased ROS, and also cancer, where phosphorylation and acetylation PTM of Cyt c may provide a mechanism to avoid apoptosis and enable progression of the disease [44,49].

**Figure 10.**
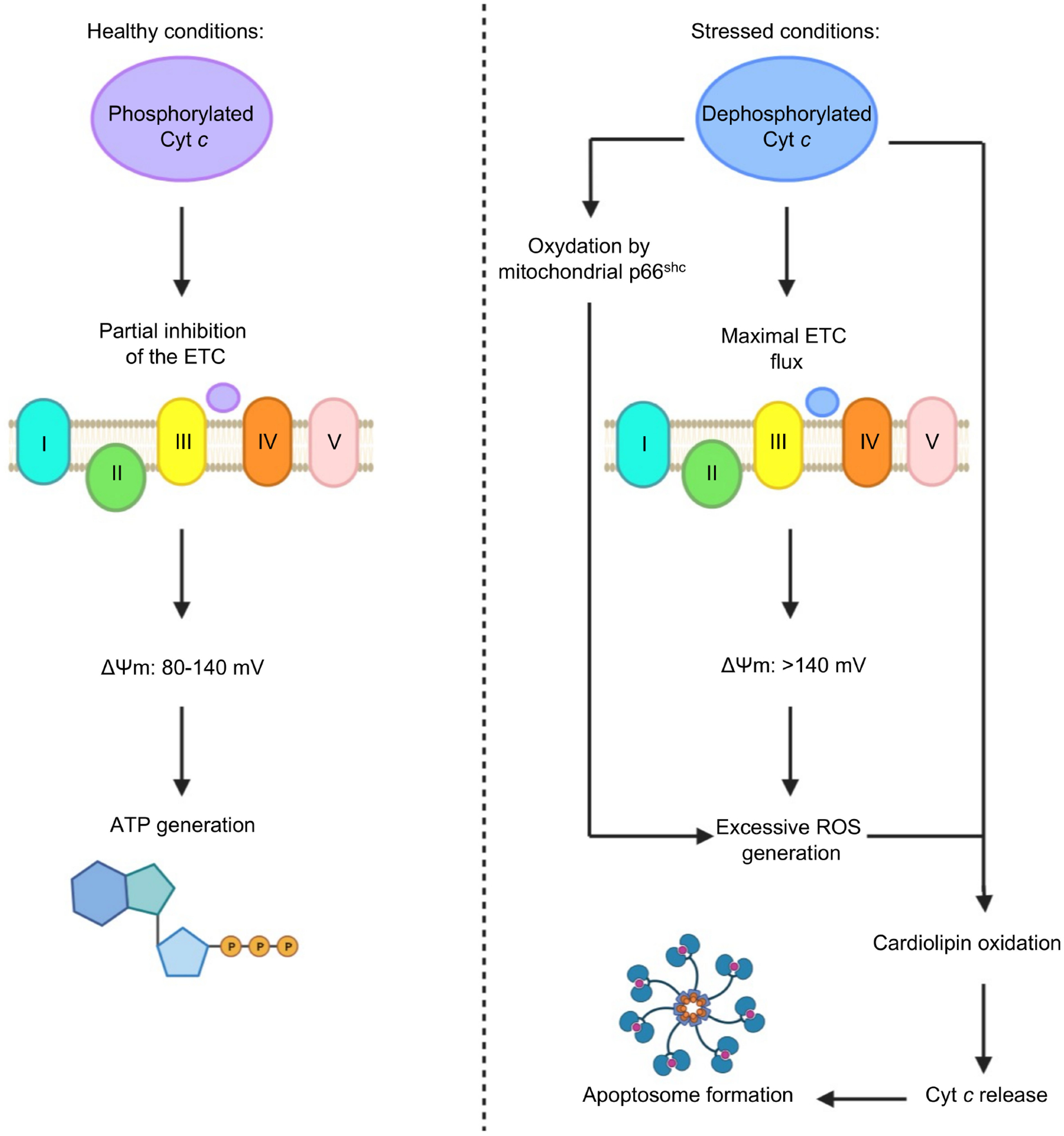
Schematic view of the effects of phosphorylated and dephosphorylated Cyt c in the mitochondria. (Image was created using BioRender.com).

Under cellular stress, Cyt c is dephosphorylated by a phosphatase and there is no ‘controlled respiration’ and maximal ETC flux is possible [39]. Respiratory control or ‘controlled respiration’ is the mechanism that regulates the activity of the ETC complexes through the mitochondrial membrane potential (ΔΨ_m_). In the case of unstressed conditions, respiratory control helps maintain the ΔΨ_m_ in a range from 80 to 140 mV. These ΔΨ_m_ levels are adequate to allow the generation of ATP-by-ATP synthase, which optimally occurs at 100 to 120 mV, and to prevent the formation of ROS. Whenever ‘controlled respiration’ is lacking, and maximal respiration rates are exhibited, there is a hyperpolarization of the ΔΨ_m_, and the levels surpass the 140 mV. This allows the formation of ROS at complexes I and III in excessive amounts due to the large quantities of electrons in the ETC. ROS, such as hydrogen peroxide, can also be formed as a result of the oxidation of dephosphorylated Cyt c by the mitochondrial pro-apoptotic protein p66^shc^ [39]. It is also important to mention that dephosphorylated Cyt c helps catalyze the oxidation of cardiolipin, which is involved in the release of Cyt c from the mitochondria; cardiolipin oxidation also requires ROS, which are readily available under these conditions. Once the dephosphorylated Cyt c is released from the mitochondria it can aid in the formation of the apoptosome [39]. The possible outcomes based on the conditions that Cyt c is exposed to are presented in Figure 10.

### 2.5. The Importance of the Heme Fe Center to Cyt c Activity

Non-Fe versions of Cyt c are widely studied for structural insight about the protein and they open a window into better understanding the function of the Fe metal in the heme group. The cobalt(III), zinc(II), and copper(II) substituted Cyt c reveal the importance of the redox potential of the metal center. Co(III) is coordinated to the Met-80 and His-18 ligands [50,51] as in the native Cyt c species and the Cu(II) is believed to be similarly coordinated [52]. There was some confusion about the exact nature of the Zn(II) coordination but a ^1^H NMR solution study elucidated the typical six-coordinate binding modality with the Met-80 and His-18 serving as axial ligands [53]. Despite the Co(III)-substituted Cyt c appearing native-like in its structure [54,55], it differs in its redox activity from Feheme [50]. The Co(III) Cyt c has an E_m,pH 7_ = −140 mV. It cannot be reduced by NADPH Cyt c reductase, microsomal or mitochondrial NADH, nor by succinate Cyt c reductase. It can, however, be oxidized by Cyt c oxidase at about 45% of the rate for native Cyt c [50]. Co(III) binding appears to alter the integrity of the reductase binding site and modify the oxidase binding site [50]. The Cu(II)- and Zn(II)-substituted Cyt c, which are also structurally similar to native Cyt c, are redox inert [52,56,57]. That said, Zn(II) Cyt c can be photo-induced to undergo electron transfer reactions [56].

While Fe redox activity is central to the function of Cyt c in the ETC, in serving as a redox agent, and in regulating ROS, it is not a requirement for its proapoptotic role. The Cu(II)- and Zn(II)-substituted Cyt c were observed to be able to induce apoptosis with about 50% potency of native Cyt c [58]. However, for optimal apoptotic activity to take place, native Cyt c has to be present in reducing conditions, such as the reducing environment found in cytoplasm [58].

## 3. Biomedical Applications of Cyt c in Cancer Research

Elucidating the biochemistry of Cyt c and the mechanism of action of the different functions that it plays has inspired great interest in its potential for real-life applications. Numerous Cyt c mutants and de novo “artificial Cyt c” maquettes have been designed as electron transport and redox functional systems [59–62]. Some of these systems employ the modification of amino acid residues to enhance specific electrochemical interactions, such as the addition of positively charged residues to enhance superoxide radical sensing [61]. Other systems utilize Cyt c as an electron mediator crucial to the formation of electroactive biofilms found in microbial fuel cells [62]. Cyt c’s bioreductive ability has also inspired the study of artificial complexes that mimic the protein to reduce toxic pollutants such as heavy metals [63]. While important, these applications are outside the scope of this review. Instead, we will focus on the promising utility Cyt c offers in cancer research as a clinical marker and therapeutic agent.

### 3.1. Cyt c as a Biomarker

As a molecule released into the extracellular space because of cell damage, Cyt c can serve as a damage-associated molecular pattern (DAMP). DAMPs are molecules that are part of the innate immune response which are released from damaged or dying cells and locate to inappropriate compartments and sites where they typically do not exist. They trigger a noninfectious inflammatory response by binding to a pattern recognition receptor (PRR), signaling the damage to help mitigate further damage. Cyt c could be useful as a biomarker of severe damage in the mitochondria or cell death [64]. Clinical studies have demonstrated that the released Cyt c ultimately enters the blood circulation. The levels of Cyt c in blood serum allow for monitoring of diseases such as multiple organ dysfunction syndrome and systemic inflammatory response syndrome; patients suffering from these conditions exhibit higher levels of Cyt c [64].

When abnormalities occur in apoptosis, a variety of diseases can result, including cancer. Afify et al. used the quantitative sandwich enzyme immunoassay technique to measure apoptotic markers in serum [65]. The serum levels of programmed cell death protein 4 (PCDC4) and Cyt c were found to increase in patients with hepatocellular carcinoma (HCC) compared to chronic hepatitis patients, which were higher compared to controls (*p* < 0.05) [65]. The correlation of Cyt c with the variables studied revealed that Cyt c was inversely correlated with alanine transaminase (ALT), and it was inversely correlated with tumor marker carcinoembryonic antigen (CEA). The mean serum values of PDCD4 and Cyt c were significantly higher in cases with a single lesion HCC. Regarding the grade of differentiation, grade II (well-differentiated) demonstrated higher mean values of Cyt c. Differentiation describes how much or how little a tumor resembles the normal tissue from which it arose. The specificity of Cyt c and PDCD4 as diagnostic markers for HCC was higher than that of serum alpha-fetoprotein, a tumor marker currently used to track different types of cancers [65].

### 3.2. Cyt c in Anticancer Therapies

Cyt c, as is the case with other important mediating proteins of apoptosis, has garnered considerable attention for use in anticancer strategies. Intrinsic apoptosis is strictly controlled by the BCL-2 family of proteins [66,67]. These proteins use several domains, including their BH1, BH2, and BH3 domains, to exert their anti or proapoptotic function [68–71]. The BH3 domain participates in proapoptotic activity. BH3-only proteins trigger apoptosis by directly interacting with BAX or BAK proteins, which results in disrupting the binding of antiapoptotic proteins bound to them. This interaction leads to the oligomerization of BAX and BAK followed by mitochondrial outer membrane permeabilization (MOMP) and ultimately the release of Cyt c into the cytosol [72,73]. Several recombinant proapoptotic proteins are being explored as anticancer drugs (Table 1). Cyt c is prominent in these efforts due to its broad spectrum of effect. A total of two categories of drug strategies involving Cyt c are discussed: small molecule drugs that induce Cyt c release from mitochondria, and novel approaches to intravenously inject Cyt c using a drug delivery vehicle.

#### 3.2.1. Cyt c as Part of the Mechanism of Small Molecule Anticancer Drugs

**Figure 11.**
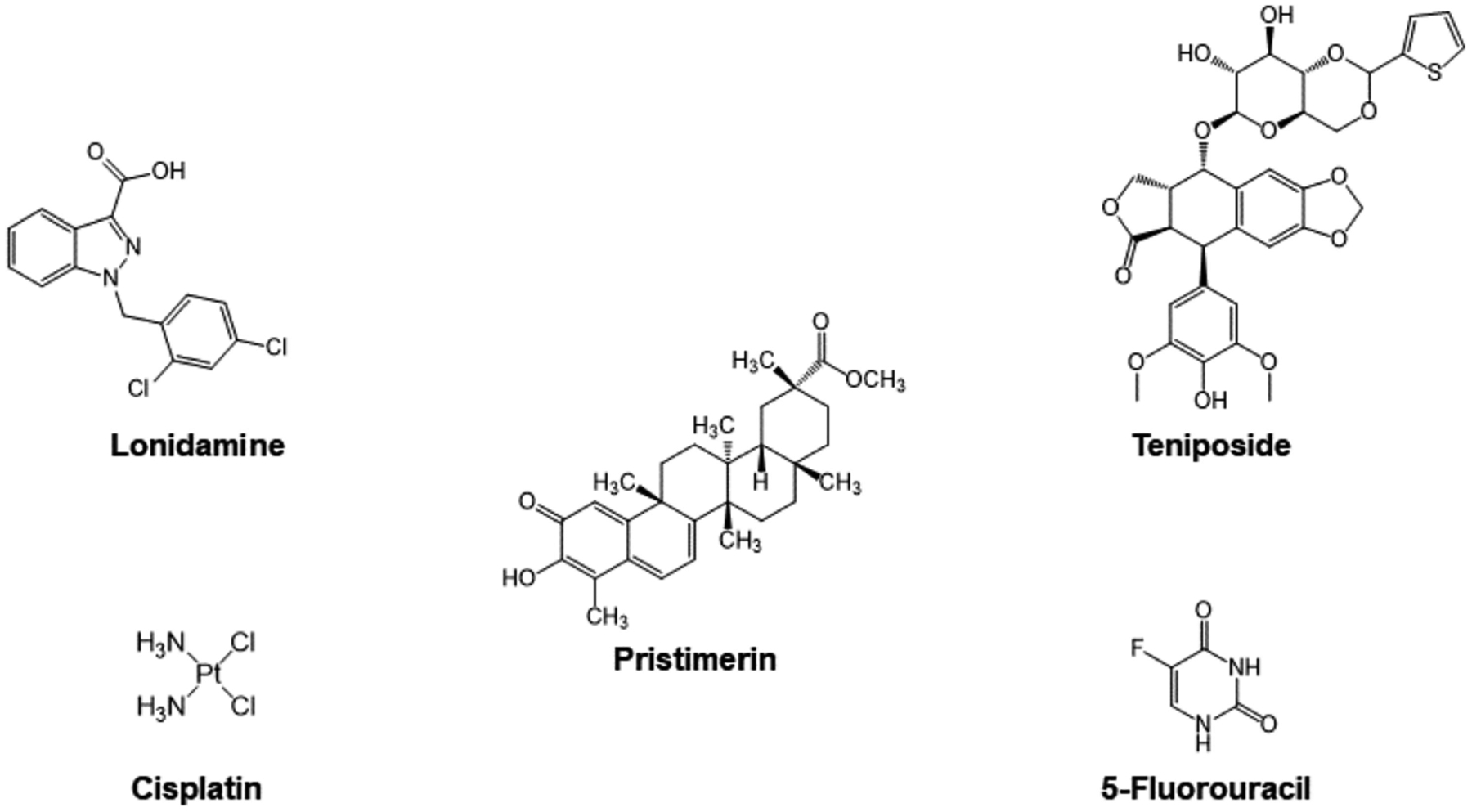
Small molecule anticancer drugs that involve Cyt c in their anticancer mechanism.

A select group of anticancer drugs function by taking advantage of powerful mechanisms already found in our bodies (Figure 11), not necessarily by the original intent of their design. Of these mechanisms, one is the induction of apoptosis. This property has led researchers to explore different methods that can weaponize Cyt c against cancer. An economic approach is to utilize the Cyt c already present in our cells. To design a compound that can effectively use Cyt c against cancer, it is necessary to review some of the drugs that have been studied for this purpose. Platinum-based metallodrugs are powerful agents in the treatment of cancer. The use of these chemotherapeutics has been correlated with the release of Cyt c in the cytosol and subsequent apoptosis [74–76]. Other drugs that involve Cyt c as part of their mechanism are organic compounds, such as lonidamine, pristimerin, teniposide and 5-fluorouoracil. Most of these drugs operate by creating structural changes in the mitochondrial membrane or by increasing the overall expression of the Cyt c. Such changes lead to an increased amount of Cyt c in the cytosol, which has been correlated to cell death. However, it is important to note that none of these drugs are selective for targeting cancer cells. In this section, we evaluate the role of Cyt c in the context of anticancer drugs, because insight into the mechanisms that allow these drugs to exploit the protein can facilitate the optimization and development of future anticancer agents.

##### Cisplatin

Over the last few decades, a large variety of Pt(II) and Pt(IV) complexes have been developed to improve cancer therapy [77]. Of these, five Pt(II) compounds have advanced to clinical use as first-line drug treatment of certain solid tumors. Cisplatin, carboplatin, and oxaliplatin were approved by the United States FDA. Nedaplatin was approved in Japan and Lobaplatin was approved in China. The compounds primarily work by forming platinum–DNA complexes that interfere with DNA replication but feature additional cellular effects including altering the mitochondrial membrane and triggering the release of Cyt c.

Cisplatin is the parent Pt(II) anticancer drug used in chemotherapy for the treatment of various types of cancer, including advanced bladder cancer and metastatic ovarian and testicular cancer. It binds to the G bases of DNA and forms crosslinks with GG sequences [77], causing DNA damage, interfering with DNA repair mechanisms, and inducing mitochondria-mediated apoptosis in cancer cells [74,76]. The initial cancer patient response to cisplatin is high. However, most patients eventually develop resistance to it. Different mechanisms have been proposed for this resistance, including changes in the cellular process due to the influence of the drug, detoxification of the drug, inhibition of apoptosis, and increased effectiveness of DNA repair. Kleih et al. investigated the role of the mitochondria in cisplatin-induced apoptosis and its molecular basis by using high-grade serous ovarian cancer (HGSC) cell lines [76]. It was observed that different cell lines are either sensitive or resistant to cisplatin, being the sensitive cell lines characterized by higher relative mitochondrial content (density of mitochondria). It was also found that glutathione (GSH) enhanced the viability of cells incubated with cisplatin. GSH is an antioxidant capable of preventing damage to cellular components caused by ROS. Furthermore, cisplatin-induced ROS production in two sensitive cell lines and one resistant cell line was analyzed showing higher ROS production (~50%) in the sensitive ones. These are ROS that are produced by mitochondria (mtROS) and can lead to oxidative damage to DNA, membranes, and mitochondrial proteins. Increased mitochondrial content was correlated with increased mtROS production and enhanced sensitivity to cisplatin-induced cell death, confirming that mitochondrial content and the resulting mitochondrial capacity to produce ROS critically determines HGSC cell sensitivity to cisplatin-induced apoptosis. A study by Gorbunova et al. examined the role of BNIP3 in cisplatin-induced apoptosis in lung adenocarcinoma cells [74]. BNIP3 is a BH3-only family proapoptotic protein that serves as a regulator of mitophagy, the selective degradation of mitochondria to prevent accumulation of dysfunctional mitochondria to maintain cells healthy. It was shown that the knockout (KO) of BNIP3 caused inhibition of oxygen consumption and stimulated production of the mtROS [74]. The suppression of BNIP3-dependent mitophagy significantly increased Cyt c release after apoptosis induction. Mitochondrial damage and reduced respiration results in ETC destabilization and electron leakage generating in mtROS. A549 BNIP3 KO cells were more susceptible to cisplatin cytotoxicity [74]. In this same study it was examined how cisplatin effects the activity of the onco-suppressor protein p53, which modulates the response to cell death stimuli. Phosphorylation of this protein at key residues is necessary for its tumor suppressive role [78]. The ratio of phosphorylated p53 to unphosphorylated p53 increased after apoptosis induction by cisplatin regardless of BNIP3. This work demonstrates the possibility of suppressing BNIP3 to bypass the tumor cell resistance to therapeutic drugs [74].

##### Organic Drugs

Lonidamine is an anticancer agent used to increase tumor sensitivity towards chemo and radiotherapy. Studies have found that its ability to enhance cell death is tied to alterations of the mitochondrial membrane [79]. Specifically, the use of lonidamine has been correlated with a drop of mitochondrial membrane potential in isolated mitochondria [79]. A steady potential of mitochondrial membrane is needed for the functioning of a healthy cell, whereas a drop in potential limits the entry of essential proteins into the mitochondria [80]. The specific mechanism by which lonidamine can lead to a decrease in mitochondrial membrane potential is tied to the opening of a permeability transition (PT) pore and swelling of the mitochondrial matrix [81]. It was also shown to enhance the pore-forming activity of the adenine nucleotide translocase (ANT) protein, an inner mitochondrial membrane protein [81]. The PT pore and ANT pathways that lonidamine can use serve as examples of how an anticancer agent could trigger Cyt c release by acting directly on transmembrane channels of the mitochondria.

Pristimerin is a quinone methide triterpenoid with antimicrobial, anti-inflammatory, antiperoxidative, and antitumor properties, extracted from plant species *Celastracea* and *Hippocrateacea*. Pristimerin’s antitumor properties are thought to be related to Cyt c release, due to a significant increase in the protein in cytosolic fractions following 3 μM drug administration (at least greater than 100% measured by Western blotting) [82]. Pristimerin (3 μM) has not been observed to generate ROS when used for the treatment of the cancer cell line MDA-MB-231, meaning that its ability to induce Cyt c release is not related to oxidative stress. Another potential apoptotic pathway that uses Cyt c release is the opening of the outer mitochondrial pores by the protein Bax [83]. However, pristimerin was not able to induce Bax expression in the same cell line. It is possible that pristimerin could be triggering Cyt c by interacting directly with the mitochondrial membrane. Pristimerin has a steroid-like structure that could facilitate its insertion into the mitochondrial membrane, thus altering its permeability. Similar behavior has been observed with digitonin, a triterpenoid compound that can induce Cyt c release by inserting itself into the outer mitochondrial membranes [82]. A different study used pristimerin in the context of the human liver cancer cell line HepG2. Pristimerin was found to induce apoptosis related to Cyt c release but coupled with a drop in mitochondrial membrane potential and the generation of ROS [83]. Pristimerin’s ability to induce Cyt c release distinctly in cell lines MDA-MB-231 and HepG2 highlights how the mechanisms of the same anticancer agent can differ in a physiological context.

Teniposide is generally used for the treatment of acute lymphocytic leukemia in children [84]. It is classified as a podophyllotoxin, which has antitumor properties due to its ability to irreversibly inhibit topoisomerase II, an enzyme that cuts DNA strands to manage tangles and supercoils [85]. Sánchez-Alcázar et al. used the MDA-MB-231 cell line to explore Cyt c release after administering teniposide [86]. An increased amount of Cyt c was observed in cytosolic fractions, coupled with an increase in cell death. However, in contrast with other drugs, Cyt c amounts also increased inside mitochondrial cell fractions. More so, no subsequent drop in mitochondrial membrane potential has been related to teniposide. Therefore, a mechanism such as that of pristimerin in the MDA-MB-231 cell line, where the drug interacts with the mitochondrial membrane directly, is plausible. An alternative explanation is that teniposide acts only indirectly on the mitochondrial membrane. The increase in Cyt c in cytosolic fractions is observed after 24 h of teniposide administration, whereas the increase in mitochondrial fractions has been observed much earlier, in a time interval suggestive of a cause-and-effect relationship between mitochondrial and cytosolic Cyt c content [86]. It is plausible that teniposide promotes a pathway that enhances the synthesis of new Cyt c to facilitate apoptosis by a different mechanism. The case of teniposide highlights how an anticancer drug can increase the release of Cyt c without interacting with the mitochondrial membrane.

Considering the different mechanisms by which small molecule anticancer drugs can stimulate Cyt c induced apoptosis, there is great promise for combinatorial therapy. Single-agent chemotherapy typically results in high toxicity, acquired drug resistance, and serious side effects because of increased drug dosages. Therefore, synergistic drugs for combinatorial therapy are sought. This has been observed with the drug 5-fluorouracil (5-FU), an antimetabolite “pyrimidine antagonist” used to treat colorectal, breast, liver, and ovarian cancer, amongst others. The drug, 5-FU, has a similar structure to the DNA base pyrimidine, therefore, it interferes with nucleoside metabolism, leading to cytotoxicity and cell death [87]. This mechanism involves the misincorporation of fluoronucleotides into RNA and DNA, causing the inhibition of the nucleotide synthetic enzyme thymidylate synthase [88]. The drug, 5-FU, has the capacity to lower the mitochondrial membrane potential and stimulate the overproduction of ROS. The combination of 5-FU with andrographolide (ANDRO), a natural bicyclic diterpenoid lactone, and selenocystine (SeC) results in potent cytotoxicity [88]. Pro-apoptotic Bax expression is involved in ANDRO-induced hepatocellular carcinoma (HCC) cell line HepG2 apoptosis. The combination of 5-FU and ANDRO enhances the conformational changes of BAX, essential to the apoptotic pathway [88]. It was observed that SeC could serve as a chemosensitizer for 5-FU. In A375 human melanoma cells, the combination of SeC and 5-FU enhanced ROS-mediated DNA damage leading to higher amounts of DNA fragmentation and nuclear condensation [89].

#### 3.2.2. Drug Delivery Systems for the Application of Cyt c as an Anticancer Biodrug

The use of proteins as therapeutics has gained increasing attention due to their unique and exploitable properties in anticancer activity [12]. Consequently, the use of proapoptotic proteins such as Cyt c, which can function as an anticancer agent, in conjunction with traditional chemotherapy strategies such as cisplatin or doxorubicin, have shown promising results [90]. Cells will naturally release Cyt c into the cytosol once a proapoptotic signal is given through the intrinsic apoptotic pathway resulting in programmed cell death. Yet, many cancer cells have become proficient at evading self-death through Cyt c suppression [91,92]. A possible strategy to overcome this suppression is to externally administer and supplement Cyt c into a patient for it to undergo its proapoptotic function. However, there are many challenges that must be addressed to implement Cyt c for cancer-treatment purposes. In general, many proteins are labile and do not easily cross the cellular membrane (Cyt c is membrane impermeable) to reach their intended therapeutic target. In vivo delivery of proteins is burdened by rapid proteolytic degradation and the delivery must be in a targeted manner to avoid systemic and off-target effects [93].

As an overview, when considering what delivery agent is appropriate for Cyt c we must consider its physical and chemical properties. Cyt c is a small protein with dimensions of 2.1 × 2.5 × 3.7 nm^3^, which equates to around 20 nm^3^. It possesses basic surface characteristics (pI: 10.8) owed to its positively charged residues lying on its solvent-exposed section. These positive charges are crucial for its activity by interacting with the terminal mitochondrial Cyt c oxidase and the Apaf-1 in the cytosol which triggers the apoptosome assembly and the apoptotic caspase cascade. A Western blot study performed on human kidney 293 and MCF7F breast carcinoma cells indicated that a comparable amount of Cyt c was found in these cells (between 40 and 100 fg/cell) [94]. This range is useful as an upper limit for endogenous releasable Cyt c and sets a ballpark range of desired drug delivery release of the protein to effectively induce apoptosis in receptive cancer cells. A microinjection (delivering material into a living cell with a micropipette or microneedle) of 40 to 400 femtoliters of a Cyt c solution (3 mg/mL) delivered ~120 fg of the protein into the 293, HeLa, and MCF7F cells [94]. The microinjected Cyt c was very effective in triggering apoptosis in the 293 cells and was moderately effective in the HeLa cells but demonstrated no activity in the MCF7F cells. A ten-fold higher amount of delivered Cyt c was equally ineffective in the MCF7F cells. The absence of Cyt c sensitivity was due to the absence of caspase 3 within MCF7 cells in general [94–96], rendering Cyt c inert at inducing apoptosis [94]. This study is extremely helpful in identifying cancer types that would be most sensitive to an anticancer regimen of Cyt c.

**Figure 12.**
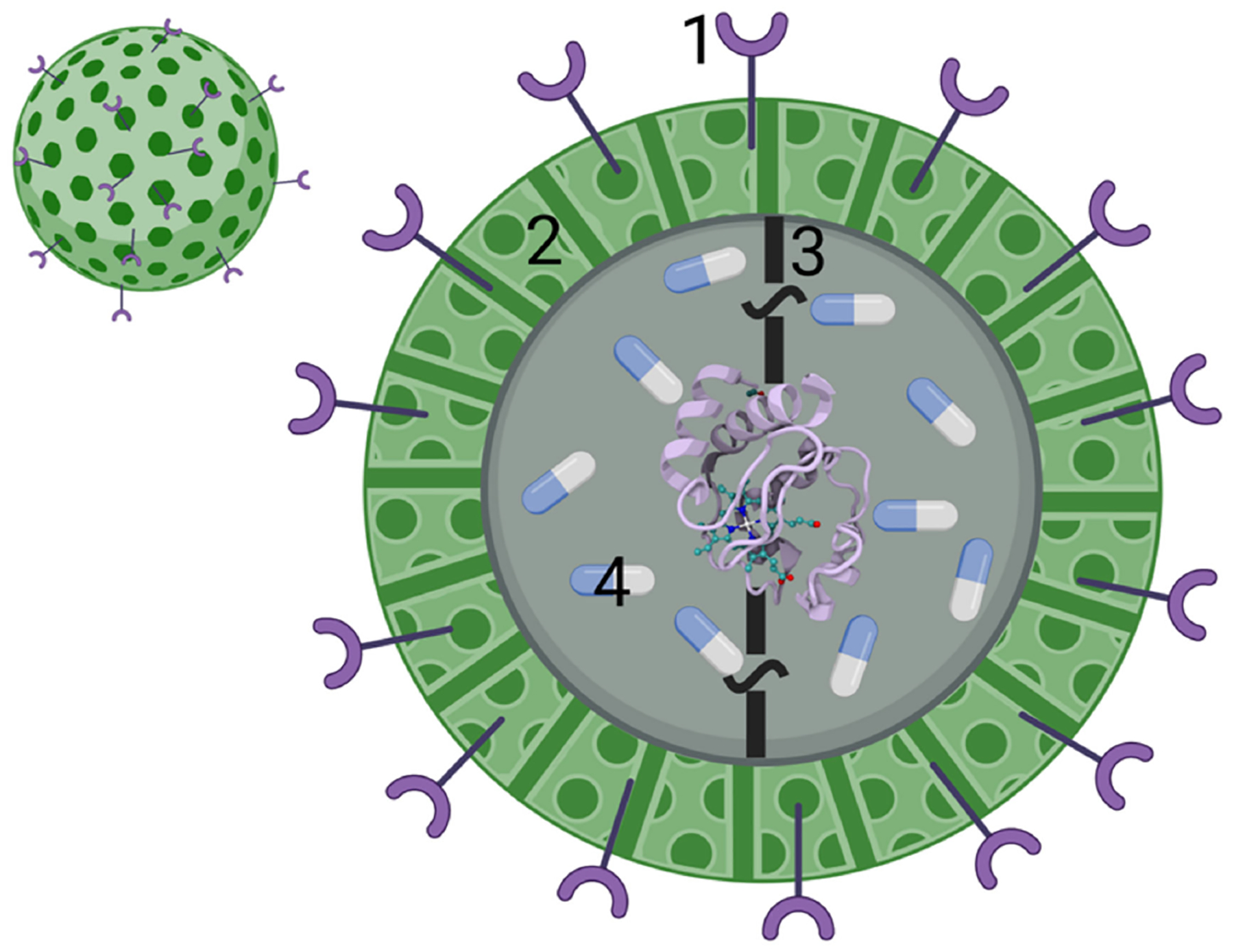
Smart DDS for delivery of Cyt c into cancer cells. Typical scaffolds consist of one or more of the following properties. 1. A cell receptor recognition moiety. 2. An encapsulation or nanoparticle formulation for exploiting the EPR effect. 3. An external stimuli response factor to release Cyt c within the cytosol. 4. Another drug for co-treatment.

Smart drug delivery systems (DDS) have been designed to maximize the payload release of drugs at target sites in a controlled manner to decrease drug toxicity and promote therapeutic efficiency [97]. To improve therapeutic indices by decreasing the lack of tumor specificity and unwanted side consequences of the typical cytotoxic drugs used in chemotherapy, researchers are focusing on nanoparticle (NP)-based and protein-based DDS technologies [98,99]. Different carrier scaffolds have been designed for Cyt c delivery into cancer cells and, herein, we will discuss a selection of these ranging from nanoparticles, Cyt c assembled particles, nanogels, and conjugation to other proteins. These scaffolds consist of one or more of the following properties (Figure 12): 1. A cell receptor recognition moiety targeting receptors overexpressed in cancer cells; 2. An encapsulation or nanoparticle formulation to take advantage of the enhanced permeation and retention effect (EPR) [100]; 3. An external stimuli response factor for releasing Cyt c in the cytosol; and 4. Another drug for co-treatment [90].

##### Nanoparticle-Based Cyt c Delivery Systems

**Figure 13.**
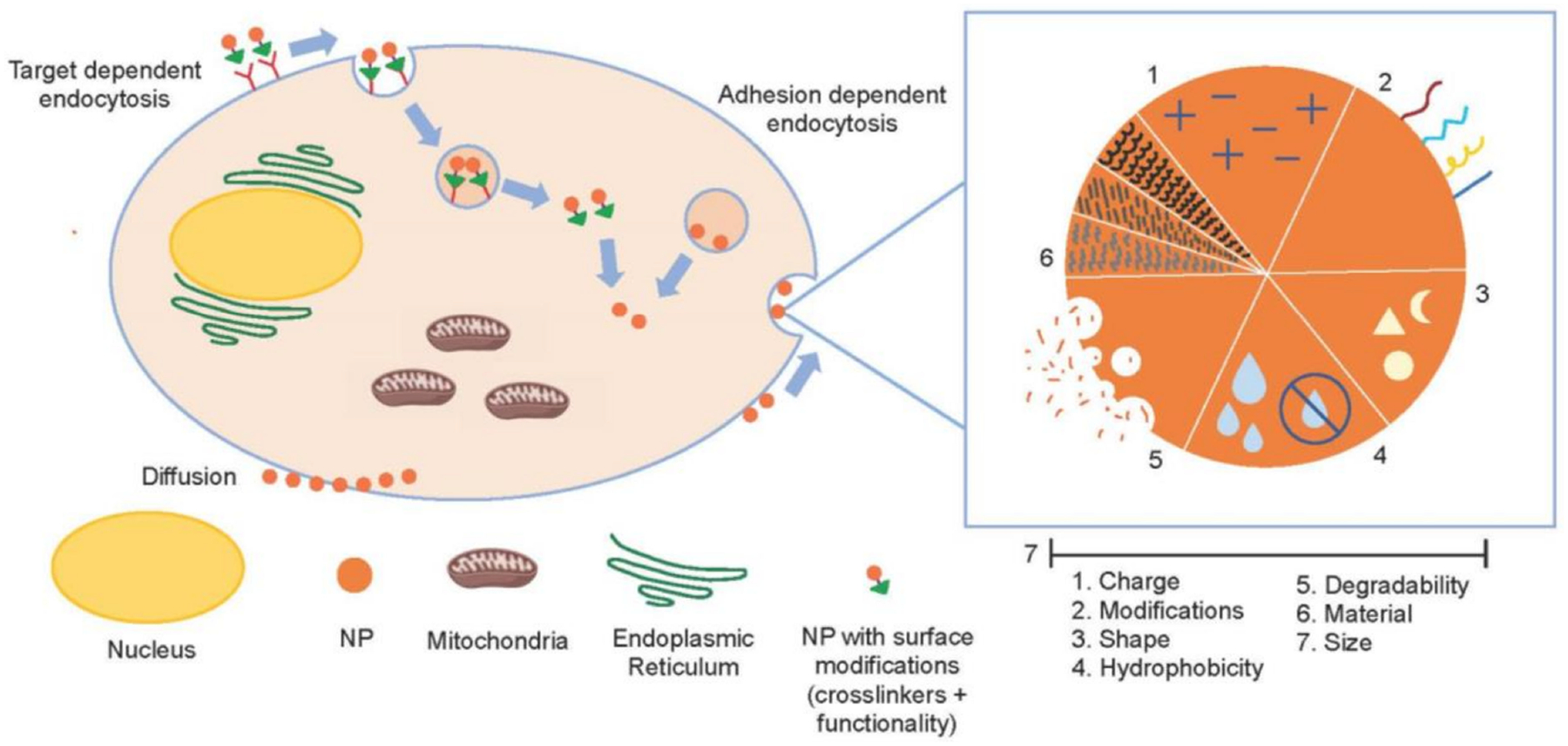
Nanoparticle cancer cell entry via different methods: target-dependent endocytosis, adhesion-dependent endocytosis or penetration, and important parameters for consideration throughout the process of nanoparticle design.

The feasibility of a NP-based DDS depends mainly on its physicochemical characteristics. Nanoparticles take on a variety of sizes, shapes, and properties, all-important at the moment of drug delivery [77]. They can interact electrostatically with cells and adhere to them, facilitating adhesion-dependent endocytosis [101]. Nanoparticles can also diffuse passively across the cellular membrane, taking advantage of the vasculature surrounding the tumor cells, which is very porous when compared to healthy cells, and enabling particles sized < 400 nm to accumulate in the cytoplasm of tumor cells after cellular uptake. Diffusion back into the bloodstream is difficult and the nanoparticles are retained within the cell. This is known as the enhanced permeation and retention effect (EPR) [100]. Ideal nanoparticles range in diameter size between 10 nm and 200 nm. Nanoparticles with a size less than 200 nm are rapidly eliminated by the kidneys and NPs greater than this diameter can result in the risk of risk immune recognition [102]. A third way nanoparticles can enter the intracellular environment is via target-dependent mechanisms [101]. Tumor cells overexpress some receptors that healthy cells have in lower quantities (e.g., hyaluronic acid (HA) receptor CD44), which makes specific targeting of these receptors a promising way to target cancerous tissue with specificity. Figure 13 depicts the different cell entry mechanisms for nanoparticles, all of which depend entirely on the design of the DDS system [103].

A variety of nanoparticle templates have been used to efficiently deliver Cyt c by itself and in combination with other drugs into cells. In some cases, more than one NP type has been incorporated into the design. The surface of several of these delivery systems has been modified to incorporate receptor targeting ligands and stimuli sensitive linkers to facilitate drug release. Herein, we will focus on a selection of the NP-based drug delivery systems designed for Cyt c to highlight key structural features.

Hamad and Aubin illustrated how the effect of ligand charge on nanoparticle surface can affect the three-dimensional structure of Cyt c [104]. Gold nanoparticles (1.5 nm diameter) were synthesized with triphenylphosphine (TPP ligands). Via ligand exchange, the NPs were modified to change the nature of the charge distribution on the NP surface with positively charged aminoethanethiol (AET), negatively charged bis-(*p*-sulfonatophenyl)phenylphosphine (BPS), and neutral poly(ethylene glycol)thiol (PEG-SH). The AET-, BPS-, and PEG-NPs were attached to yeast or horse Cyt c by incubation overnight at 25 °C. Circular dichroism (CD) spectroscopy was used to evaluate the structure of the Cyt c in the three conjugates. The neutral ligand did not change the protein structure but the charged ligands did cause denaturation to the protein particularly in the vicinity of the C102 amino acid as a consequence of electrostatic interactions [104].

NP DDS strategies will try to use adsorption as a means for delivery. Utilizing mesoporous silica nanoparticles (MSN) is an attractive strategy due to their mechanical properties, facile surface modification, and biocompatibility. Traditionally, MSNs are not capable of storing protein guests due to small pore sizes (2–3 nm in diameter), yet recently work has been conducted in the creation of large-pore mesoporous silica nanoparticles (LPMSNs). Choi et al. synthesized enlarged-pore MSNs through hydrolytic surface erosion of the O–Si–O bond, effectively forming a silanol surface (^−^O–Si–O) [105]. The resulting LPMSNs showed variance in pore distribution with a range of 2–10 nm pores, average particle size of ~74 nm, and a notable change in surface charge from neutral to negatively charged. Both characteristics increased the encapsulation of Cyt c two-fold from 12.2 wt% to 24.4 wt%. Yet their current formulation has a direct, guest-solvent interaction, allowing for premature protein leakage at different pH values. To overcome this, Guo et al. [106] have synthesized pH-responsive LPMSNs using ethyl-acetate hydrolyzed tetraethoxysilane that have a pore-size correlated to the amount of ethyl-acetate added with a range from 10–30 nm in diameter. By grafting the silane coupling agent, DMMA-APTES (containing an acid-sensitive amide bond) on the LPMSN surface, they could obtain a negative surface charge, depending on the pH of the solution, to allow for AuNP pore-capping once the protein guest was introduced. They reported a protein loading capacity of about 9.6 wt%, utilizing this strategy that may be attributed to the absence of the negative pore charge, yet the capping AuNPs significantly obstructs protein leakage. These studies illustrate that MSNs are a promising scaffold for protein delivery, which pore size and charge play a crucial role in allowing effective protein encapsulation where negative pore charges tend to favor stronger interactions from the positively charged Cyt c surface.

Méndez et al. presented another MSN system in which glycosylated Cyt c was stabilized and immobilized [107]. Cyt c was glycosylated with lactose to stabilize the protein and minimize its degradation upon chemical immobilization. To ensure an efficient protein discharge inside the cancerous cell, a stimulus-responsive controlled release system with redox-sensitive disulfide bonds was incorporated to release the apoptotic protein under the reducing intracellular conditions but not under extracellular conditions. By attaching the lactose to Cyt c before the attachment of the disulfide linker, the structure of the protein was not altered according to the CD spectra and other spectroscopic characterization [107].

Combinatorial approaches for NP-based drug delivery systems are also being developed. Al-Shakarchi et al. employed a method consisting of immobilizing Cyt c onto the surface of hybrid iron oxide-gold nanoparticles (HNPs) while simultaneously supplementing the cells with clinically used anticancer agents (e.g., doxorubicin, paclitaxel, oxaliplatin, vinblastine, and vincristine) for liver cancer therapy [90]. Using this experimental design, the researchers would be able to pinpoint if the chemotherapeutic drugs and HNP DDS simultaneously administered could result in greater efficiency in increasing the levels of apoptosis within the cell lines that would lead to cellular death. Evidence of uptake was reported on the following cell lines used: hepatocellular carcinoma (HepG2), epithelial hepatoma (Huh-7D), and endothelial hepatocellular carcinoma (SK-hep-1). After confirming the entry of the HNP-Cyt c into the cells, the cytotoxic effect was evaluated. The caspase-3 levels for HNP-Cyt c were tested for apoptosis detection. Combinatorial treatments in HepG2 and Huh-D7 gave increased levels of caspase-3 when compared to the single-drug treatment, presumably due to possessing a synergistic effect on the tumor cells. The SK-hep-1 cell line proved to be highly resistant, as no significant effects were obtained.

Morales et al. presented a novel NP-based delivery of Cyt c in which the nanoparticle core consisted of Cyt c itself [108]. For stabilization, the Cyt c NP was coated with the biocompatible, biodegradable, and non-toxic hydrophobic polymer poly(lactic-co-glycolic) acid (PGLA). A redox-sensitive response factor was incorporated via a hetero-bifunctional linker (succinimidyl 3-(2-pyridyldithio) propionate). After the smart release of the Cyt c NP, apoptosis was induced. The integrity of the soluble protein after nanoprecipitation was compared with native Cyt c and was found to be around 90%. SEM images revealed a spherical shape of the NPs and confirmed the particle size to be in a nm range (diameter: 80–150 nm), ideal for particles with expected passive delivery properties [108]. To improve on this design, Morales et al. added a folate-receptor targeting amphiphilic copolymer (FA-PEG-PLGA-SH) attached to Cyt c through a redox-sensitive bond [109]. The FA-Cyt c NP size could be fine-tuned from ~250 nm to ~350 nm [109,110]. The modification retained excellent stability under extracellular physiological conditions but released the Cyt c NPs in the intracellular reducing environment, resulting in potent cytotoxicity [109]. The Cyt c NPs exhibit high caspase 3 activity (80–96%) [109,110]. The FA-Cyt c NPs were examined in C57BL/6 mice implanted with GL261 glioma tumor [109]. The drug was infused at 1 μL/h, 100 mg/mL over 3 days. Control mice were infused with saline solution. The tumor size in the mice treated with FA-Cyt c NPs was 40% smaller compared to the control mice. Tumor tissue and healthy tissue examined one day after the termination of treatment revealed significant apoptosis only in the tumor area [109]. In a related study, it was determined that the route of entry of the FA-Cyt c NPs into the GL261 cells was the proton-coupled folate transporter (PCFT) [111]. The NPs could cause cell death in glioma cells according to the expression level of PCFT. It was also observed that FA-Cyt c NPs could target the folic acid receptor alpha expressing Lewis Lung Carcinoma (LLC) cell line and was three times more selective against the tumor cells than normal cells in an LLC mouse model [110].

Nanogels are another formulation for NP-based drug delivery of Cyt c. They are nanoparticles composed of hydrogels or a crosslinked hydrophilic polymeric network. Crosslinked synthetic polymers or biopolymers are an effective and commonly used strategy for drug delivery and other biomedical applications. Using nanogels as a delivery agent for Cyt c shows great potential because of the vast options of polymers that can be made to maximize protein loading and control protein release. Zhong et al. showed how redox-sensitive HA nanogels (HA-NGs) can be used via intravenous administration for a targeted delivery of Cyt c into MCF7 human breast cancer cells that were inoculated into female nude mice [112]. The Cyt c–HA–NG complex was formed through reverse nanoprecipitation of hyaluronic acid-graft-oligo ethylene glycol-tetrazole, then in the presence of L-cystine dimethacrylamide it can be readily photo irradiated and crosslinked. This crosslinking results in a nanogel with unifying disulfide bonds, which are redox-responsive. The nanogel formulation offered high-yield protein loading with 40.6 wt% of Cyt c, which may be attributed to the high-density negative charged of the hyaluronic acid in the nanogel scaffold. The Cyt c was rapidly released upon the nanogel interaction with cytosolic glutathione, attributing a controlled release mechanism. Furthermore, it showed no systemic cytotoxic effect whilst inhibiting tumor growth through high-tumor penetration and specificity through high CD44 receptor recognition. What is unclear about this study was how Cyt c was able to display antiproliferative activity against the MCF7 cells, given their deficient caspase 3 expression [94–96]. Taking into consideration that the nanogels alone demonstrated no activity, could it be that the Cyt c–HA–NG complex facilitated an alternative apoptotic or other cytotoxic mechanism?

In total, smart nanomaterials are a fascinating strategy that unify many desirable characteristics from delivery systems, which can be synthetically tailored to the delivery of Cyt c. There are, of course, limitations in the application of these materials. Despite the existence of the EPR effect, only ~5% of an administered NP-drug typically ends up in the tumor, given that their localization is a random diffusional process [113]. All NPs have known stability issues and associated toxicity due to their breakdown, for such reasons as thrombosis and the release of metal ions [77]. These stability and toxicity factors are important to consider in the engineering of a NP delivery system of Cyt c.

##### Protein-Based Cyt c Delivery Systems

An interesting form of Cyt c delivery is using other peptide and protein species. Transport peptides and proteins have the characteristics of being highly resistant to degradation by enzymes, confer high drug stability and excellent in vivo distribution. Protein delivery systems rely on two strategies; either bioconjugation through a covalent and reversible linker or assembly around the protein of interest.

Macone et al. utilized a chimeric ferritin nanovehicle to target human promyelocytic leukemia cells (NB4 cells) [114]. The chimeric ferritin was composed of a human and Archaeglobus fulgidus ferritin, which allowed assembly capabilities while maintaining biocompatibility. Ferritin proteins possess a cage-like structure with a hollow (350 nm^3^) cavity. They modified 50% of the internal cysteine residues with S-carboxymethyl groups to increase the negative internal surface charge, increasing, as seen previously, the electrostatic interactions between the ferritin and Cyt c. Ferritin assembly was conducted by dissociating ferritin in low ionic strength buffer then reconstituting with a MgCl_2_ solution, allowing reassembly of the 24-meric structure. Cyt c was then encapsulated by ferritin with a protein loading of 1.5 units of Cyt c per unit of ferritin. NB4 cells were treated with the ferritin–Cyt c assemblies presented with a targeted delivery owed to the recognition of ferritin by the CD71 receptor involved in the transferrin-iron uptake cell mechanism. The cells showed high levels of fluorescently labeled Cyt c being liberated into the cell cytosol and inducing apoptosis [114].

Yeh et al. made use of galactosylated bovine serum albumin (BSA–GaSM) conjugated to Cyt c through a reducible disulfide linkage [115]. Because galactosamine is recognized by asialogycoprotein receptors (ASGPR), the human hepatocarcinoma cell lines HepG2 and Hep3B were selected for this study because of the overexpression of ASGPR, while Mahlavu cells, which do not express ASGPR, were used as a negative control. Yeh et al.’s method offered a targeted delivery mechanism while conferring stability and cell permeability through receptor mediated endocytosis. However, results showed that Cyt c–BSA–GaSM conjugates were able to enter all cell lines and induce apoptotic cell death. The lack of specificity might be due to other carbohydrate-recognizing receptors expressed on all of the cells. Using chemistry analogous to the work of Yeh et al. [115], Saxena et al. synthesized a soluble transferrin (Tf)–Cyt c conjugate [116]. Cancer cells have an overexpression of transferrin receptors (TfRs) compared to healthy cells due to their increased demand for iron, as it is a vital metal for cell proliferation. The Tf–Cyt c conjugate was tested in different cancer cell lines (A549, HeLa, and K562 cells) and in the noncancer cell line MRC5. The conjugate demonstrated an expected antiproliferative effect against the A549 and HeLa cells, but not the MRC5 cells. However, no antiproliferative effect was observed against the K562 cells, which is believed to be due to the mitochondrial apoptosis pathway being blocked in these cells, inhibiting mitochondrial Cyt c release and even caspase activation after Cyt c release [117].

Chemical conjugation of Cyt c to protein transporters can result in heterogeneous mixtures with a low yield of the conjugate of interest. To circumvent this problem, Delinois et al. recently prepared a soluble recombinant hybrid protein consisting of Cyt c and the cell-penetrating peptide chlorotoxin [118]. This recombinant soluble conjugate was easy to synthesize using bacterial protein production machinery, resulting in a relatively high yield and homogeneous 1 Cyt c: 1 chlorotoxin conjugate with potent cytotoxicity and favorable therapeutic index against glioma cells. Structural integrity and apoptotic activity of Cyt c were maintained in the hybrid protein [118]. This approach of generating recombinant peptide/protein-Cyt c conjugates opens a new avenue for the drug delivery of proapoptotic proteins.

## 4. New Insight for Optimizing Cyt c Anticancer Drug Design

By evaluating the biochemical mechanisms by which Cyt c functions in cells to facilitate cell survival and death and assessing the different efforts to apply it as a vital agent for cancer care, this work serves to elucidate optimal approaches to bring Cyt c to the anticancer drug market. Several smart DDS demonstrate the feasibility of achieving improved cancer cell selectivity via receptor targeting and the needed high drug payload through cellular stimuli release. The challenges of advancing these designs to clinical use are the complex synthetic steps and associated costs and possible lack of homogeneity, stability, and reproducibility of drug delivery composition and drug loading. Another major challenge is ensuring that the DDS composition is compatible with the physicochemical properties of Cyt c and does not deter its stability and activity. Molecular biology recombinant technology offers the potential to streamline the smart DDS-drug compound synthesis and to facilitate product synthesis scalable to industrial levels. However, more research is needed in how to build in all necessary parameters (receptor recognition and stimuli release, etc.) into the recombinant approach.

Other valuable factors to consider are how to increase the potency and longer-term use of Cyt c. A Cyt c mutant has been identified, displaying enhanced apoptotic activity relative to the native variant and exhibiting generally low toxicity to the human body [32]. Mutant variants such as this one should be engineered that maximize cell death capability while retaining low toxicity, which can be aided with an appropriate smart DDS. It is clear that certain cancer types will not be receptive to Cyt c drug treatment; nonetheless, Cyt c could work as a generally broad-spectrum drug. It is thus imperative to evaluate how to administrate it in a drug regimen. Given the heterogenous nature of cancer cells within different cancer types, a combinatorial approach to cancer treatment is often sought [90], even when combined drugs or approaches may not be synergistic [119]. The use of inhibitors of select apoptotic regulatory proteins could boost the potency of Cyt c as observed for cisplatin [74]. Some commonly used drugs already work by employing Cyt c as part of their mechanism. For these drugs, Cyt c could work as a second line treatment, especially if they induce high acquired resistance. Resistance to Cyt c is also a very relevant issue. While smart DDS can help with overcoming Cyt c suppressive mechanisms, the recently recognized biochemical pathway of anastasis complicates Cyt c drug design [92,120]. Anastasis (Greek origin meaning “rising to life”) describes the recovery of dying cells after being at the brink of death, and appears to be an intrinsic recovery phenomenon against apoptosis-induced cell death and possibly other cell death mechanisms [92]. Tang et al. found that late-stage apoptosis was reversed in HeLa cells, brain cells, and cervical cancer, among others [120]. These cells revealed a startingly higher presence of permanent oncogenic transformations. This finding could explain why some cancers that seem to have reached remission sometimes result in a re-diagnosis later, in some cases being characterized as more aggressive forms of cancer. The application of Cyt c would thus have to be carefully considered to prevent a resistance response to it as an endogenous inducer of apoptosis.

## Figures and Tables

**Table 1.** Recombinant proapoptotic proteins explored as anticancer drugs. Some content is Reprinted from Trends in Biotechnology, 36, Serna, N.; Sánchez-García, L.; Unzueta, U.; Díaz, R.; Vázquez, E.; Mangues, R.; Villaverde, A., 318–335, Copyright (2018), with permission from Elsevier [12].

Protein	Source	Mechanism of Action	Recombinant Protein (Producing Organism)	Cancer Tested
BID	Homo sapiens	Activator: interacts with high affinity to all antiapoptotic proteins and directly activates BAX and BAK	*E. coli*	Breast, ovarian, and prostate cancer
PUMA	Homo sapiens	Activator: interacts with high affinity to all antiapoptotic proteins and directly activates BAX and BAK	*E. coli*	Colon cancer
BAD	Homo sapiens	Sensitizer: interacts with antiapoptotic protein BCL-2 and BCL-XL with high affinity	*E. coli*	Glioma, leukemia, and gastrointestinal carcinoma
BIK	Homo sapiens	Sensitizer: interacts with antiapoptotic protein BCL-2 and BCL-XL with high affinity	*E. coli*	Colon adenocarcinoma
BAKBH3	Homo sapiens	Antagonizes antiapoptotic protein function	*E. coli*	Cervical and colon cancer
Cyt c	Homo sapiens	Trigger apoptosis by interacting with Apaf-1 and cleave procaspase-9	*E. coli*	Many cancer types
